# Tomography of the subducting Pacific slab and the 2015 Bonin deepest earthquake (Mw 7.9)

**DOI:** 10.1038/srep44487

**Published:** 2017-03-15

**Authors:** Dapeng Zhao, Moeto Fujisawa, Genti Toyokuni

**Affiliations:** 1 Department of Geophysics, Tohoku University, Sendai 980-8578, Japan

## Abstract

On 30 May 2015 an isolated deep earthquake (~670 km, Mw 7.9) occurred to the west of the Bonin Islands. To clarify its causal mechanism and its relationship to the subducting Pacific slab, we determined a detailed P*-*wave tomography of the deep earthquake source zone using a large number of arrival-time data. Our results show that this large deep event occurred within the subducting Pacific slab which is penetrating into the lower mantle. In the Izu-Bonin region, the Pacific slab is split at ~28° north latitude, i.e., slightly north of the 2015 deep event hypocenter. In the north the slab becomes stagnant in the mantle transition zone, whereas in the south the slab is directly penetrating into the lower mantle. This deep earthquake was caused by joint effects of several factors, including the Pacific slab’s fast deep subduction, slab tearing, slab thermal variation, stress changes and phase transformations in the slab, and complex interactions between the slab and the ambient mantle.

The 30 May 2015 Bonin earthquake is the deepest large event occurring in and around Japan during the observational history of the Japan Meteorological Agency (JMA) in the past 140 years^1^. Globally, it is the deepest event with M ≥ 7.8 in the seismological record^2^. It is an isolated event locating over 100 km deeper than the Wadati-Benioff zone seismicity recorded so far (Fig. 1). Hence, this Bonin event is not part of the mainstream deep seismicity, and its relationship to the subducting Pacific slab and the mantle transition zone (MTZ) is unclear. It is important to clarify the precise source location of this deep event relative to the subducting slab, which will shed new light on the deep slab structure and subduction dynamics.

To date, several studies have investigated this unusual deep earthquake but obtained controversial results on its source location relative to the subducting Pacific slab. Ye *et al*.^2^ investigated the source rupture process of this deep event by inverting its teleseismic waveforms, and suggested two models of the Bonin slab related to the 2015 deep event. One model shows that this deep event took place within a folded slab continuous along strike. The other model shows that the deep event occurred within a torn/buckled slab that is recumbent to the north but steeply dipping to the south, and this event could also be in a detached piece of slab from earlier subduction^2^. Takemura *et al*.^3^ examined seismogram envelopes of high-frequency P waveforms of the 2015 Bonin deep earthquake and its aftershock (Mw 5.6) recorded by the dense seismic networks on the Japan Islands, and suggested that the 2015 deep event was located at the bottom of the stagnant Pacific slab in the MTZ, rather than in the middle or the upper parts of the slab where deep earthquakes usually take place. Porritt and Yoshioka^4^ used teleseismic receiver functions to study the mantle structure down to a depth of ~800 km beneath a seismic station above the 2015 Bonin deep event. Their results indicate that the base of the MTZ is at a depth of 690 km, i.e., ~10–20 km below the hypocenter of the 2015 deep event, and there are significant P-to-S wave conversions in and below the MTZ. Based on their receiver-function results, Porritt and Yoshioka^4^ proposed that the Pacific slab is piling up at the base of the MTZ and the 2015 deep event occurred in a deep limb of the slab immediately above the base of the MTZ.

Seismic tomography is an effective tool for investigating the three-dimensional (3-D) structure of the Earth’s interior, in particular, for clarifying the morphology and structure of subducting slabs e.g. refs 5 and 6. High-resolution tomographic imaging can also reveal the detailed structure of large earthquake source zones, providing important information on the rupture nucleation processes e.g. ref. 5. In this work we apply a multiscale tomographic method^5^,^6^ to abundant P-wave arrival-time data recorded by world-wide seismic stations (see Supplementary Figure S1) including those from the dense seismic networks in Japan and East China (Fig. 2) to determine a high-resolution mantle tomography beneath the Izu-Bonin region. Our tomographic results reveal the subducting Pacific slab clearly, which shed new light on the causal mechanism of the 2015 Bonin deep earthquake and subduction dynamics in the study region.

## Data

**Figure 2:**
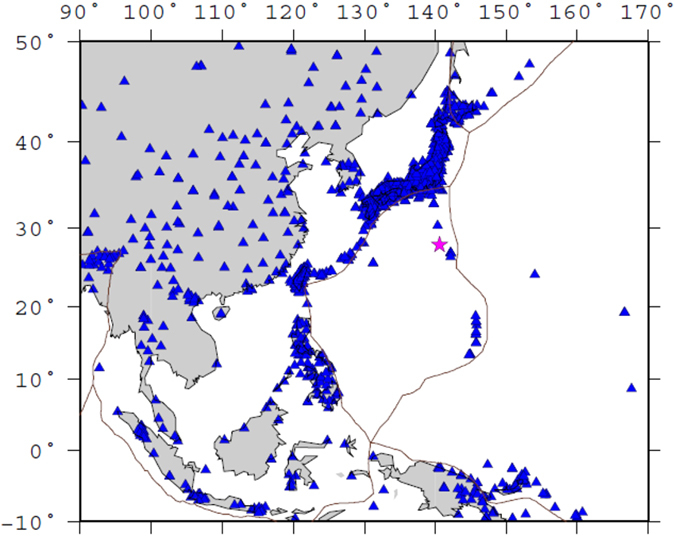
Seismic stations in the study region. The red star denotes the epicenter of the 2015 Bonin deep earthquake. The brown lines denote plate boundaries. This figure was generated using the Generic Mapping Tools^58^ version 4.5.8 (http://gmt.soest.hawaii.edu).

We used arrival-time data of earthquakes which are selected from the repressed ISC (International Seismological Center) data base^7^, the JMA Unified Earthquake Catalogue^1^, and the Annual Bulletin of Chinese Earthquakes^8^. For selecting a best set of earthquakes, the crust and mantle (0 to 700 km depth) are divided into cubic blocks. The block size is 1° × 1° × 20 km for the whole globe but 0.5° × 0.5° × 10 km for the target Izu-Bonin region (see Supplementary Figure S2). Among the many events located within each block, only one earthquake is selected which was recorded by the largest number of seismic stations and has the smallest error in its hypocenter location. Our data set thus collected contains 5,126,696 arrival times of P, pP, PP, PcP and Pdiff waves from 39,323 earthquakes recorded by 9141 seismic stations in the world (see Supplementary Figure S1). Hence the target Bonin region (Fig. 2 and Supplementary Figure S2) is well sampled by the up-going and down-going rays of both the direct P waves and later phases^6^,^9^.

## Results

**Figure 3:**
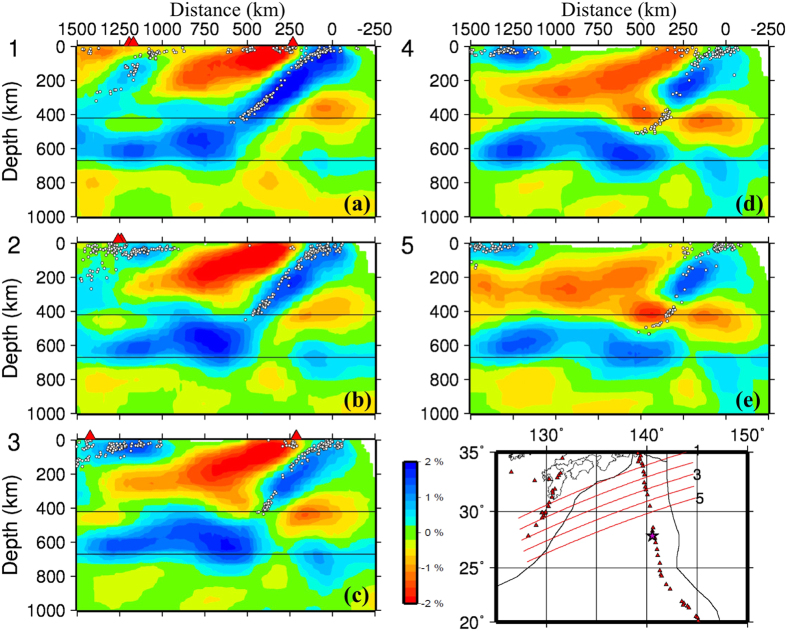
Vertical cross-sections of P-wave tomography along the profiles shown in the inset map. Low and high velocity perturbations are shown in red and blue colors, respectively, whose scale (in %) is shown beside (**c**). Active volcanoes are shown in red triangles. The white circles denote seismicity within a 30-km width of each profile. The two black lines represent the 410 and 670 km discontinuities. The red star in the inset map denotes the epicenter of the 2015 Bonin deep earthquake. The horizontal distance is from the Izu-Bonin trench. This figure was generated using the Generic Mapping Tools^58^ version 4.5.8 (http://gmt.soest.hawaii.edu).

**Figure 4:**
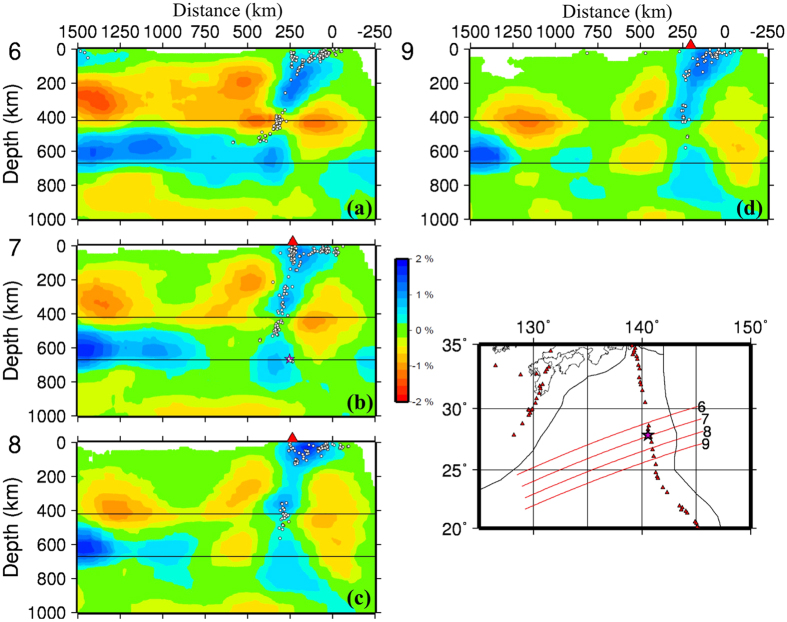
Vertical cross-sections of P-wave tomography. The same as Fig. 3 but for four vertical cross-sections in the Bonin region. The red star in (**b**) denotes the hypocenter of the 2015 Bonin deep earthquake. This figure was generated using the Generic Mapping Tools^58^ version 4.5.8 (http://gmt.soest.hawaii.edu).

**Figure 5:**
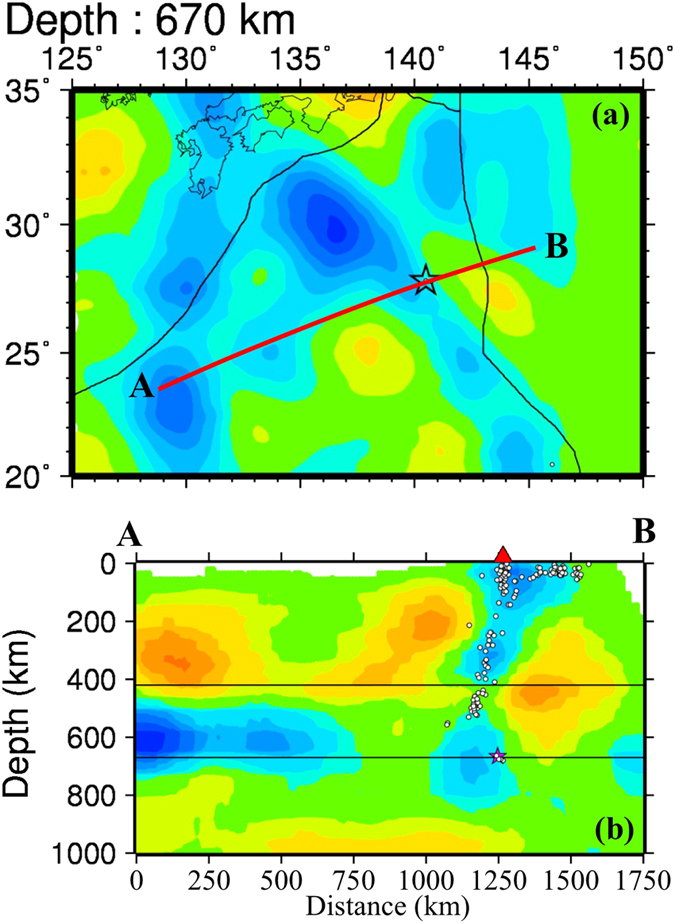
P-wave tomography in the source zone of the 2015 Bonin deep earthquake. (**a**) Map view at 670 km depth. (**b**) Vertical cross-section along the profile A-B shown in (**a**). The other labeling is the same as that in Fig. 3. This figure was generated using the Generic Mapping Tools^58^ version 4.5.8 (http://gmt.soest.hawaii.edu).

Figures 3 and 4 show vertical cross-sections of P-wave velocity (Vp) tomography beneath the Izu-Bonin region obtained by this study. Map views of the Vp tomography are shown in Supplementary Figure S3. The local seismicity and active arc volcanoes^10^ are also shown in the tomographic images. Prominent dipping high-velocity (high-V) zones are revealed in the upper mantle down to a depth of ~410 km, which reflect the subducting Pacific and Philippine Sea slabs in which intermediate-depth and deep earthquakes occur actively (Figs 3 and 4). In Fig. 3d,e, the Pacific slab with deep seismicity shows a lower velocity around 410 km depth, which is an artifact due to a lower resolution of the tomographic image there. The active arc volcanoes are located above low-velocity (low-V) zones in the mantle wedge above the subducting slabs. The low-V zones represent the source areas of arc magmatism and volcanism caused by a combination of fluids from the slab dehydration and corner flow in the mantle wedge driven by the plate subduction e.g. refs 11, 12, 13, 14. The subducting Philippine Sea slab is also imaged clearly to west of the Ryukyu Trench (Figs 3 and 5a and Supplementary Figure S3), indicating that the Philippine Sea slab has also reached the MTZ depth, being consistent with previous local and regional tomographic models e.g. refs 13, 15, 16, 17.

In the vertical cross-sections north of ~28°N latitude (Figs 3 and 4a), the Pacific slab becomes flat in the MTZ. South of ~28°N latitude (Fig. 4b–d), however, the Pacific slab becomes quite steep and is penetrating into the lower mantle. The high-V slab image is quite clear in and around the source zone of the 2015 Bonin deep earthquake (Figs 4 and 5). The large-scale features of our tomography are consistent with those of the previous global and regional tomographic models in the Izu-Bonin region e.g. refs 6, 15, 16, 17, 18, 19, 20, 21, 22, but the slab image in the source zone of the 2015 Bonin deep earthquake (Fig. 5) is different from those of the previous tomographic models (see Supplementary Figure S4), which are discussed in the next section.

The 2015 Bonin deep event is relocated using our 3-D Vp model. The relocated focal depth is 667.2 ± 0.5 km (see Supplementary Table S1), and the relocated hypocenter is located within the high-V slab but close to the eastern boundary of the near-vertical slab (Fig. 5). Note that the hypocenter is just the initial point of the earthquake rupture. Waveform inversions show that the average source depth of this great deep event is ~680 km and its overall source dimension is ~40 km in a shallowly-dipping fault plane^2^.

Our whole-mantle tomographic images beneath the Izu-Bonin region are shown in Supplementary Figure S5. Below the Pacific slab in the MTZ, intermittent high-V zones appear in the lower mantle, and broad high-V anomalies exist above the core-mantle boundary, which reflect old pieces of the Pacific slab that have collapsed down to the lower mantle and reached the core-mantle boundary due to gravitational instability caused by phase transformations e.g. refs 6 and 21.

Detailed resolution analyses are made to confirm the main features of the tomographic results (see Supplementary Information Figures S6–S11). The results of these resolution tests show that main features of the tomographic results (Figs 3, 4, 5), in particular, those in and around the source zone of the 2015 Bonin deep earthquake, are quite robust.

## Discussion

**Figure 6:**
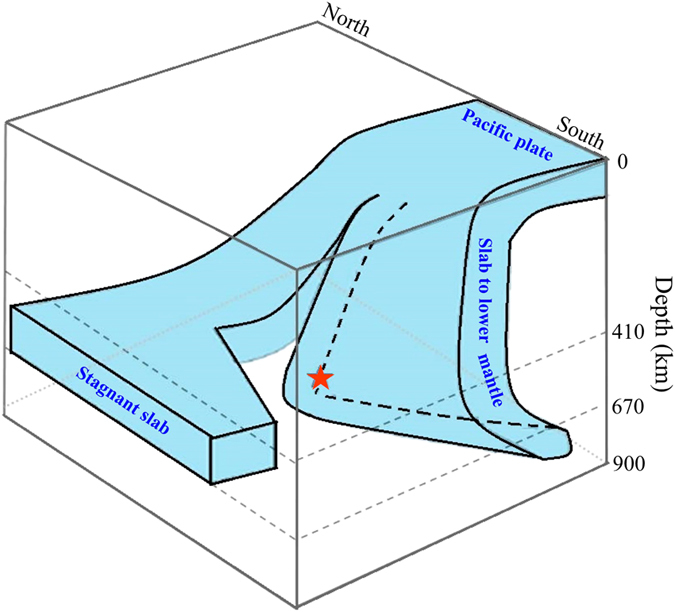
A schematic diagram of the subducting Pacific slab beneath the Bonin region. The red star denotes the hypocenter of the 2015 Bonin deep earthquake.

Based on the present results and also previous seismic studies of the Izu-Bonin region e.g. refs 16, 17, 18, 19, 20, 21, 22, 23, we propose a model on the subducting Pacific slab and the source location of the 2015 Bonin deep earthquake in the slab (Fig. 6). The Pacific slab is split at ~28° N latitude, i.e., slightly north of the hypocenter of the 2015 deep event which occurred at 27.7° N latitude (see Supplementary Table S1). In the north, the slab becomes stagnant in the MTZ, whereas in the south the slab is directly penetrating into the lower mantle. Miller *et al*.^18^,^19^ showed that the Pacific slab has an anomaly of physical properties in the upper mantle, which is located in an area north of the 2015 Bonin hypocenter. They found that most of the intermediate-depth and deep earthquakes occurring within this anomaly in the slab had lateral tension mechanisms, potentially related to the slab tearing. The 2015 Bonin deep event also had a lateral tension mechanism, which may be related to the slab tear^3^.

Our model (Fig. 6) is similar to the second model of Ye *et al*.^2^. In other words, our tomographic results do not support their first model but their second model, because a simple vertical high-V zone is clearly visible in the 2015 source area (Fig. 5b), which reflects the Pacific slab penetrating into the lower mantle. We agree with Takemura *et al*.^3^ on their point that the 2015 Bonin deep event occurred within the slab and close to the lower slab boundary, but we do not prefer their model which shows a flat slab in the MTZ at the 2015 Bonin hypocenter. The slab pileup model^4^ is similar to the first model of Ye *et al*.^2^ and is even more complex, which is quite different from our model (Fig. 6) and our tomographic images (Fig. 5) which show a simple slab geometry without buckling. The receiver-function image^4^ might be over-interpreted, which was obtained beneath only one seismic station. The conversion points in and below the MTZ in the receiver-function image^4^ may not represent boundaries of a buckled slab but complex phase changes in the slab at the MTZ depths.

To date, several studies of receiver-functions and waveform modeling have shown that the 670-km seismic discontinuity is depressed down to ~690 km depth beneath the Bonin region^4^,^24^,^25^,^26^. The focal depth of the 2015 Bonin deep event was estimated to be in a range of 664–682 km (see Supplementary Table S1). Hence, it is almost certain that this deep event occurred within the MTZ. The slab-related high-V zone looks thicker at depths of 600–800 (Fig. 5b), which may suggest that the slab has thickened near the MTZ bottom due to resistance at the upper-lower mantle boundary e.g. refs 6, 21 and 27.

Previous 3-D velocity models of the Izu-Bonin-Mariana region are obtained by using global tomography or large-scale regional tomography e.g. refs 6, 16, 17, 18, 19, 20, 21, 22, which generally have a lower resolution because large blocks or grid intervals were adopted in the 3-D model parameterization. Because there are few seismic stations in this region, the tomographic models are sensitive to the data set, model parameterization, inversion algorithm, and damping and smoothing parameters adopted in the inversion. Hence the relationship between the 2015 Bonin deep event and the subducting slab is not clear in the previous tomographic models (e.g., Supplementary Figure S4). In contrast, our present study focuses on the source zone of the 2015 Bonin deep event, and we use a much better data set and adopt a dense 3-D grid in the target region, hence our present tomographic model shows a clear relation between the slab and the deep event (Fig. 5). To better resolve the detailed structure of the subducting slab in the MTZ, P and S waves reflected and/or converted at the MTZ discontinuities e.g. refs 26, 28, 29, 30 should be collected and used in tomographic imaging, in addition to deploying a dense network of ocean bottom seismometers in the study region.

Many previous studies have revealed tears in subducting slabs in the upper mantle and slab tear-related intermediate-depth seismicity in many regions e.g. refs 31 and 32. In addition to the Izu-Bonin region, a slab tear in the MTZ was detected beneath western Japan^33^,^34^. Slab buckling and folding in the MTZ have been observed in several regions, which provide an important control on the distributions and focal mechanisms of deep-focus earthquakes e.g. ref. 35.

The physical processes that permit the occurrence of deep earthquakes are still not well understood^36^. Several mechanisms for deep earthquakes have been proposed, including transformational faulting triggered by metastable olivine transforming to spinel in the cold, stressed core of the subducting slab e.g. refs 27, 37, 38, 39, 40, thermal instability and run-away shear melting e.g. refs 41, 42, 43, and dehydration embrittlement e.g. refs 44, 45, 46. All of these mechanisms depend on the thermal structure of deep slabs and the deviatoric stress conditions associated with the slabs impinging on the upper-lower mantle boundary e.g. refs 2 and 43. It is challenging to distinguish between these possible mechanisms for deep earthquakes, because of the difficulty to clarify their source dimensions and rupture processes, as well as the fine slab structure in and around the source zones of deep earthquakes. Among these mechanisms, dehydration embrittlement is considered to operate for the intermediate-depth earthquakes but may not for the deep earthquakes e.g. refs 27 and 36. The viability of transformational faulting as a mechanism for deep earthquakes hinges on the presence of a sufficient amount of metastable phase^36^. So far, a metastable olivine wedge (MOW) has been detected within the subducting Pacific slab at the MTZ depths beneath Southwest Japan^47^,^48^, Mariana^49^,^50^, and the Japan Sea^51^, and it is considered that the generation of deep earthquakes in these regions is related to the existence of MOW in the slab. Hence, transformational faulting is a conventional and popular mechanism for deep earthquakes. However, our present tomography could not image the MOW in the Pacific slab, due to the limited resolution of the tomographic model and because the MOW, if it exists, should be very thin at the great depth. Waveform inversions suggest that localized stress concentration associated with the pronounced deformation of the Izu-Bonin slab and proximity to the 670-km phase transition may have played a dominant role in generating this significant earthquake^2^.

Meng *et al*.^52^ suggested that the 2013 Okhotsk deep earthquake (Mw 8.3, 610 km depth) was affected by thermal thinning of the Pacific slab because it occurred near the northern end of the slab as revealed by seismic tomography^53^. We think that the generation of the 2015 Bonin deep earthquake could be also affected by thermal variation of the slab, because this event occurred near the northern edge of the near-vertical segment of the Pacific slab (Fig. 6) where the slab must be heated by the hot ambient mantle.

Based on the above discussions, we deem that the generation of the 2015 Bonin deep earthquake was caused by joint effects of several factors, including the fast deep subduction of the Pacific slab, slab tearing and its related thermal variation, stress changes and phase transformations in the slab near the upper-lower mantle boundary, as well as complex interactions between the subducting slab and the ambient mantle. It is not clear why the isolated deep earthquake is so large, but its large size indicates the presence of a large volume of material still in seismogenic conditions^54^,^55^. There may be very infrequent mineral transformation or volatile release processes that occur only under particularly high deviatoric stress conditions allowing large dynamic stress relaxations to take place^2^.

## Methods

We conducted tomographic inversions for 3-D Vp structure using a modified version of the multiscale tomographic method^5^,^6^. For expressing the 3-D Vp structure, a denser 3-D grid with a lateral grid interval of ~50 km is arranged in a depth range of 0–1000 km beneath the target Izu-Bonin region including the 2015 Bonin deep earthquake, whereas a coarse grid with a lateral grid interval of ~220 km is arranged in the whole crust and mantle of the Earth (see Supplementary Figure S2). Vp perturbations at every grid nodes from the one-dimensional iasp91 velocity model^56^ are taken to be unknown parameters. The Vp perturbation at any location in the crust and mantle is computed by a linear interpolation of the Vp perturbations at the 8 grid nodes adjacent to that location. A 3-D ray tracing technique^11^,^21^ is adopted to calculate theoretical travel times and ray paths. The LSQR algorithm^57^ with smoothing and damping regularizations is applied to solve the large and sparse system of observational equations^5^,^11^. We conducted many tomographic inversions of our data set to search for the optimal smoothing and damping parameters considering the balance between the travel-time residual reduction and the norm of the obtained 3-D Vp model^5^,^6^,^9^.

## Additional Information

**How to cite this article**: Zhao, D. *et al*. Tomography of the subducting Pacific slab and the 2015 Bonin deepest earthquake (Mw 7.9). *Sci. Rep.* **7**, 44487; doi: 10.1038/srep44487 (2017).

**Publisher's note:** Springer Nature remains neutral with regard to jurisdictional claims in published maps and institutional affiliations.

## Supplementary Material

Supplementary Information

## Figures and Tables

**Figure 1:**
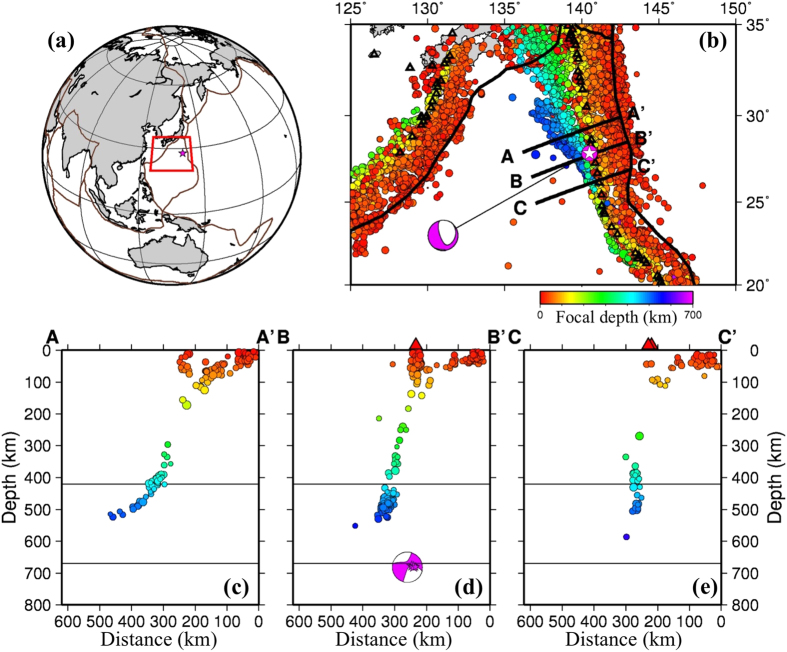
Seismicity of the study region. **(a)** The red star shows the epicenter of the 30 May 2015 Bonin deep earthquake (Mw 7.9). The red box shows the target area of the present study. The brown lines denote plate boundaries. **(b)** Seismicity of the study region from the reprocessed ISC catalogue^7^. The colors denote the focal depth whose scale is shown below (b). The black lines denote plate boundaries. The open black triangles show active volcanoes^10^. The white star and the beach ball denote the epicenter and focal mechanism of the 2015 Bonin deep earthquake. **(c–e)** Vertical cross-sections of seismicity within a width of 30 km along each of the three profiles shown in (b). Active volcanoes are shown in red triangles. The overall features of the deep seismicity in the Bonin region are consistent with those shown in Ye *et al*.^2^. This figure was generated using the Generic Mapping Tools^58^ version 4.5.8 (http://gmt.soest.hawaii.edu).

## References

[b1] Okada Y. . Recent progress of seismic observation networks in Japan – Hi-net, F-net, K-NET and KiK-net. Earth Planets Space 56, xv–xxviii (2004).

[b2] Ye L. . The isolated ~680 km deep 30 May 2015 Mw 7.9 Ogasawara (Bonin) Islands earthquake. Earth Planet. Sci. Lett. 433, 169–179 (2016).

[b3] Takemura S., Maeda T., Furumura T. & Obara K. Constraining the source location of the 30 May 2015 (Mw 7.9) Bonin deep-focus earthquake using seismogram envelopes of high-frequency P waveforms: Occurrence of deep-focus earthquake at the bottom of a subducting slab. Geophys. Res. Lett. 43, 4297–4302 (2016).

[b4] Porritt R. & Yoshioka S. Slab pileup in the mantle transition zone and the 30 May 2015 Chichi-jima earthquake. Geophys. Res. Lett. 43, 4905–4912 (2016).

[b5] Zhao D. Multiscale Seismic Tomography. Springer, pp. 304 (2015).

[b6] Zhao D., Yamamoto Y. & Yanada T. Global mantle heterogeneity and its influence on teleseismic regional tomography. Gondwana Res. 23, 595–616 (2013).

[b7] Engdahl E. Application of an improved algorithm to high precision relocation of ISC test events. Phys. Earth Planet. Inter. 158, 14–18 (2006).

[b8] Tian Y., Zhao D., Sun R. & Teng J. Seismic imaging of the crust and upper mantle beneath the North China Craton. Phys. Earth Planet. Inter. 172, 169–182 (2009).

[b9] Fujisawa M. A study of the slab structure and the 2015 large deep earthquake beneath the Izu-Bonin region. Master’s thesis, Tohoku University, pp. 118 (2016).

[b10] Simkin T. & Siebert L. Volcanoes of the World. Geoscience Press, pp. 1–368 (1994).

[b11] Zhao D., Hasegawa A. & Horiuchi S. Tomographic imaging of P and S wave velocity structure beneath northeastern Japan. J. Geophys. Res. 97, 19909–19928 (1992).

[b12] Iwamori H. & Zhao D. Melting and seismic structure beneath the northeast Japan arc. Geophys. Res. Lett. 27, 425–428 (2000).

[b13] Liu X. & Zhao D. P and S wave tomography of Japan subduction zone from joint inversions of local and teleseismic travel times and surface-wave data. Phys. Earth Planet. Inter. 252, 1–22 (2016).

[b14] Horiuchi S. & Iwamori H. A consistent model for fluid distribution, viscosity distribution, and flow-thermal structure in subduction zone. J. Geophys. Res. 121, 3238–3260 (2016).

[b15] Huang J. & Zhao D. High-resolution mantle tomography of China and surrounding regions. J. Geophys. Res. 111, B09305 (2006).

[b16] Wei W., Xu J., Zhao D. & Shi Y. East Asia mantle tomography: New insight into plate subduction and intraplate volcanism. J. Asian Earth Sci. 60, 88–103 (2012).

[b17] Wei W., Zhao D., Xu J. & Wei F. P and S wave tomography and anisotropy in Northwest Pacific and East Asia: Constraints on stagnant slab and intraplate volcanism. J. Geophys. Res. 120, 1642–1666 (2015).

[b18] Miller M., Kennett B. & Lister G. Imaging changes in morphology geometry, and physical properties of the subducting Pacific plate along the Izu–Bonin–Mariana arc. Earth Planet. Sci. Lett. 224, 363–370 (2004).

[b19] Miller M., Kennett B. & Toy V. Spatial and temporal evolution of the subducting Pacific plate structure along the western Pacific margin. J. Geophys. Res. 111, B02401 (2006).

[b20] Jaxybulatov K., Koulakov I. & Dobretsov N. Segmentation of the Izu-Bonin and Mariana slabs based on the analysis of the Benioff seismicity distribution and regional tomography results. Solid Earth 4, 59–73 (2013).

[b21] Zhao D. Global tomographic images of mantle plumes and subducting slabs: insight into deep Earth dynamics. Phys. Earth Planet. Inter. 146, 3–34 (2004).

[b22] Obayashi M. . Finite frequency whole mantle P wave tomography: Improvement of subducted slab images. Geophys. Res. Lett. 40, 5652–5657 (2013).

[b23] Okino K., Ando M., Kaneshima S. & Hirahara K. The horizontally lying slab. Geophys. Res. Lett. 16, 1059–1062 (1989).

[b24] Wicks C. & Richards M. A detailed map of the 660-kilomater discontinuity beneath the Izu-Bonin subduction zone. Science 261, 1424–1427 (1993). 17745352 10.1126/science.261.5127.1424

[b25] Castle J. & Creager K. Topography of the 660-km seismic discontinuity beneath Izu-Bonin: Implications for tectonic history and slab deformation. J. Geophys. Res. 103, 12511–12527 (1998).

[b26] Collier J., Helffrich G. & Wood B. Seismic discontinuities and subduction zones. Phys. Earth Planet. Inter. 127, 35–49 (2001).

[b27] Kirby S., Stein S., Okal E. & Rubie D. Metastable mantle phase transformations and deep earthquakes in subducting oceanic lithosphere. Rev. Geophys. 34, 261–306 (1996).

[b28] Zheng Y., Lay T., Flanagan M. & Williams Q. Pervasive seismic wave reflectivity and metasomatism of the Tonga mantle wedge. Science 316, 855–859 (2007). 17431138 10.1126/science.1138074

[b29] Contenti S., Gu Y., Okeler A. & Sacchi M. Shear wave reflectivity imaging of the Nazca-South America subduction zone: Stagnant slab in the mantle transition zone? Geophys. Res. Lett. 39, L02310 (2012).

[b30] Gu Y., Okeler A. & Schultz R. Tracking slabs beneath northwestern Pacific subduction zones. Earth Planet. Sci. Lett. 331–332, 269–280 (2012).

[b31] Meighan H., ten Brink U. & Pulliam J. Slab tears and intermediate-depth seismicity. Geophys. Res. Lett. 40, 1–5 (2013).

[b32] Vargas C. & Mann P. Tearing and breaking off of subducted slabs as the result of collision of the Panama arc-Indenter with northwestern South America. Bull. Seismol. Soc. Am. 103, 2025–2046 (2013).

[b33] Obayashi M., Yoshimitsu J. & Fukao Y. Tearing of stagnant slab. Science 324, 1173–1175 (2009). 19478177 10.1126/science.1172496

[b34] Kennett B. & Furumura T. Tears or thinning? Subduction structures in the Pacific plate beneath the Japanese Islands. Phys. Earth Planet. Inter. 180, 52–58 (2010).

[b35] Myhill R. Slab buckling and its effect on the distributions and focal mechanisms of deep-focus earthquakes. Geophys. J. Int. 192, 837–853 (2013).

[b36] Houston H. Deep earthquakes. Second Edition. Treatise on Geophysics, vol. 4. Elsevier, pp. 329–354 (2015).

[b37] Kirby S. Localized polymorphic phase-transformations in high-pressure faults and applications to the physical-mechanism of deep earthquakes. J. Geophys. Res. 92, 13789–13800 (1987).

[b38] Green H. & Burnley P. A new self-organizing mechanism for deep-focus earthquakes. Nature 341, 733–737 (1989).

[b39] Wiens D., McGuire J. & Shore P. Evidence for transformational faulting from a deep double seismic zone in Tonga. Nature 364, 790–793 (1993).

[b40] Green H. Shearing instabilities accompanying high-pressure phase transformations and the mechanics of deep earthquakes. Proc. Natl. Acad. Sci. USA 104, 9133–9138 (2007). 17468397 10.1073/pnas.0608045104 PMC1890459

[b41] Ogawa M. Shear instability in a viscoelastic material as the cause of deep-focus earthquakes. J. Geophys. Res. 92, 13801–13810 (1987).

[b42] Kanamori H., Anderson D. & Heaton T. Frictional melting during the rupture of the 1994 Bolivian earthquake. Science 279, 839–842 (1998). 9452378 10.1126/science.279.5352.839

[b43] Karato S., Riedel M. & Yuen D. Rheological structure and deformation of subducted slabs in the mantle transition zone: implications for mantle circulation and deep earthquakes. Phys. Earth Planet. Inter. 127, 83–108 (2001).

[b44] Silver P. . Rupture characteristics of the deep Bolivian earthquake of 9 June 1994 and the mechanism of deep-focus earthquakes. Science 268, 69–73 (1995). 17755232 10.1126/science.268.5207.69

[b45] Meade C. & Jeanloz R. Deep-focus earthquakes and recycling of water into the Earth’s mantle. Science 252, 68–72 (1991). 17739075 10.1126/science.252.5002.68

[b46] Omori S., Komabayashi K. & Maruyama M. Dehydration and earthquakes in the subducting slab: empirical link in intermediate and deep seismic zones. Phys. Earth Planet. Inter. 146, 297–311 (2004).

[b47] Iidaka T. & Suetsugu D. Seismological evidence for metastable olivine inside a subducting slab. Nature 356, 593–595 (1992).

[b48] Kawakatsu H. & Yoshioka S. Metastable olivine wedge and deep dry cold slab beneath southwest Japan. Earth Planet. Sci. Lett. 303, 1–10 (2011).

[b49] Kaneshima S., Okamoto T. & Takenaka H. Evidence for a metastable olivine wedge inside the subducted Mariana slab. Earth Planet. Sci. Lett. 258, 219–227 (2007).

[b50] Kubo T., Kaneshima S., Torii Y. & Yoshioka S. Seismological and experimental constraints on metastable phase transformations and rheology of the Mariana slab. Earth Planet. Sci. Lett. 287, 12–23 (2009).

[b51] Jiang G., Zhao D. & Zhang G. Detection of metastable olivine wedge in the western Pacific slab and its geodynamic implications. Phys. Earth Planet. Inter. 238, 1–7 (2015).

[b52] Meng L., Ampuero J. & Burgmann R. The 2013 Okhotsk deep-focus earthquake: Rupture beyond the metastable olivine wedge and thermally controlled rise time near the edge of a slab. Geophys. Res. Lett. 41, 3779–3785 (2014).

[b53] Jiang G., Zhao D. & Zhang G. Seismic tomography of the Pacific slab edge under Kamchatka. Tectonophysics 465, 190–203 (2009).

[b54] Lundgren P. & Giardini D. Isolated deep earthquakes and the fate of subduction in the mantle. J. Geophys. Res. 99, 15833–15842 (1994).

[b55] Okal E. “Detached” deep earthquakes: are they really? Phys. Earth Planet. Inter. 127, 109–143 (2001).

[b56] Kennett B. & Engdahl E. Traveltimes for global earthquake location and phase identification. Geophys. J. Int. 105, 429–465 (1991).

[b57] Paige C. & Saunders M. LSQR: An algorithm for sparse linear equations and sparse least squares. ACM Trans. Math. Soft. 8, 43–71 (1982).

[b58] Wessel P. & Smith W. New, improved version of Generic Mapping Tools released. Eos Trans. AGU 79, 579 (1998).

