# Supplementary material for: Tomography of the subducting Pacific slab and the 2015 Bonin deepest earthquake (Mw 7.9)
Source: Sci Rep. 2017 Mar 15;7:44487. doi: 10.1038/srep44487 (PMC5353660; doi:10.1038/srep44487)
Supplement: Supplementary Information [file srep44487-s1.pdf]

Tomography of the subducting Pacific slab and the 2015 Bonin deepest earthquake (Mw 7.9)

<u>Supplementary Information</u>

Dapeng Zhao, Moeto Fujisawa, Genti Toyokuni
Department of Geophysics, Tohoku University, Sendai 980-8578, Japan

Correspondence to: Dapeng Zhao (zhao@tohoku.ac.jp)

Abstract

This supplementary information contains the following materials: distribution of global earthquakes and seismic stations used in this work (Figure S1), the target study region and the three-dimensional (3-D) grid arranged for tomographic inversions (Figure S2), map views of the obtained P-wave tomography (Figure S3), comparison with previous tomographic models (Figure S4), vertical cross-sections of whole-mantle tomography beneath the target study region (Figure S5), results of detailed resolution tests (Figures S6-S11), and hypocentral parameters of the 30 May 2015 Bonin deep earthquake (Mw 7.9) determined by JMA, USGS and the present study (Table S1 and Figure S4).

Seismic stations and earthquakes used

Figure S1 shows epicentral distribution of 39,323 earthquakes and 9141 seismic stations, which generated 5,126,696 arrival-time data of P, pP, PP, PcP and Pdiff waves used in the tomographic inversion.

3-D grid for tomographic inversion

Figure S2 displays a map view and an east-west vertical cross-section showing the 3-D grid nodes arranged for the tomographic inversion. In the target source zone of the 2015 Bonin deep earthquake (Mw 7.9), a denser 3-D grid with a lateral grid interval of 50 km is arranged, whereas a coarser 3-D grid with a lateral grid interval of 220 km is set up in the surrounding crust and mantle of the Earth.

Map views of P-wave tomography

Figure S3 shows map views of the obtained P-wave tomography at 24 depth slices in the crust and mantle down 1300 km depth beneath the target study region. The background seismicity, major plate boundaries, and the epicenter of the 2015 Bonin deep earthquake are also shown in the tomographic images. For this optimal 3-D velocity model, the root-mean-square (RMS) travel-time residual is reduced to 1.19 s from its initial value (1.60 s) before the tomographic inversion.

Comparison with previous models

Figure S4 shows previous P-wave tomographic models at 670 km depth and along a vertical cross-section determined by Obayashi et al. (2013) and Zhao et al. (2013), which can be compared with the P-wave tomographic model obtained by the present study (Fig. 5). The hypocentral locations of the 2015 Bonin deep earthquake determined by JMA, USGS and this study (Table S1) are also shown for comparison.

Whole-mantle tomography

Figure S5 shows five vertical cross-sections of the present P-wave tomographic model down to the core-mantle boundary (CMB) beneath the Izu-Bonin region. High-velocity anomalies are visible in the lower mantle down to the CMB, reflecting old pieces of the Pacific slab collapsing down to the CMB (Zhao et al., 2013).

Resolution tests

We made detailed resolution analyses to confirm the main features of the tomographic results (Figs. 3-5 and Fig. S3). Three kinds of resolution tests are conducted. The first is a checkerboard resolution test (Fig. S6), the second is a synthetic test (Figs. S7 and S8), and the third is a restoring resolution test (Figs. S9 and S10).

To perform the checkerboard resolution test (Zhao et al., 1992, 2013) for the P-wave velocity (Vp) tomography, we first create an input checkerboard model by assigning Vp perturbations of ±3% alternatively at the 3-D grid nodes (the upper panels in Fig. S6). Then we calculate synthetic travel-time residuals for the checkerboard model with the same numbers of seismic stations, events and ray paths as those in the real data set (Fig. S1), and then we invert the synthetic data to obtain an output model (lower panels in Fig. S6). The test results show that the input checkerboard model is generally recovered in and around the subducting Pacific slab, in particular, at the mantle transition zone (MTZ) depths (410-670 km) and in the upper part of the lower mantle (700-950 km depths) (Fig. S6).

The procedures of the synthetic test (Figs. S7 and S8) and the restoring resolution test (Figs. S9 and S10) are the same as that of the checkerboard resolution test, except for the input model. For the synthetic test (Figs. S7 and S8), the input model contains the subducting Pacific slab and the slab is assumed to become flat in the MTZ. The test results show that the input slab is well reconstructed, though slight smearing occurs in the uppermost lower mantle, and slight low-velocity anomalies show up in the crust and the upper-mantle wedge above the slab, which are caused by the imperfect ray coverage and crisscrossing in those areas.

For the restoring resolution test (Figs. S9 and S10), the input model contains the

main features of the obtained tomographic model (Figs. 3-5 and Fig. S3). The test results show that the high-velocity subducting Pacific and Philippine Sea slabs and the low-velocity anomalies above and below the slabs are all recovered very well, except for some slight smearing.

The arrival-time data used in this work were all recorded by short-period seismometers installed in different regions of the world compiled by the ISC (International Seismological Center; http://www.isc.ac.uk/), the JMA (http://www.jma.go.jp) and the China Earthquake Network Center (http://www.ceic.ac.cn/), and so their picking errors are generally small, being ~0.1 to ~0.3 s and certainly smaller than 0.5 s. The arrival-time data with the best quality are selected for this work, and the data with a poor quality are all removed. We have conducted many tomographic inversions to investigate the effects of the data picking errors on the tomographic results. Before each inversion, random errors with a standard deviation ranging from 0.1 s to 0.5 s are added to the synthetic arrival-time data set calculated for the input model. The test results (Fig. S11) show that the effects of the random noise are very small, and the input models are generally well recovered, in particular, at the MTZ depths. This is because we have used a huge amount of arrival-time data (> 5 millions), and so the effects of the picking errors are greatly reduced.

The results of these resolution tests show that the main features of the tomographic results, in particular, those in and around the source zone of the 2015 Bonin deep earthquake, are quite robust.

References

Obayashi, M. et al. Finite frequency whole mantle P wave tomography: Improvement of subducted slab images. *Geophys. Res. Lett.* **40**, 5652-5657 (2013).

Zhao, D., Hasegawa, A. & Horiuchi, S. Tomographic imaging of P and S wave velocity structure beneath northeastern Japan. *J. Geophys. Res.* **97**, 19909-19928 (1992).

Zhao, D., Yamamoto, Y. & Yanada, T. Global mantle heterogeneity and its influence on teleseismic regional tomography. *Gondwana Res.* **23**, 595-616 (2013).

Table S1. Hypocentral parameters of the 30 May 2015 Bonin deep earthquake (Mw 7.9) determined by the Japan Meteorological Agency (JMA), the United States Geological Survey (USGS) and the present study.

	Origin time (H:M:S)	Latitude (degree)	Longitude (degree)	Focal depth (km)
JMA	11:23:02.2	27.960	140.680	682.00
USGS	11:23:02.0	27.840	140.490	664.00
This study (error)	11:23:02.0 (0.05)	27.740 (0.006)	140.590 (0.008)	667.20 (0.51)

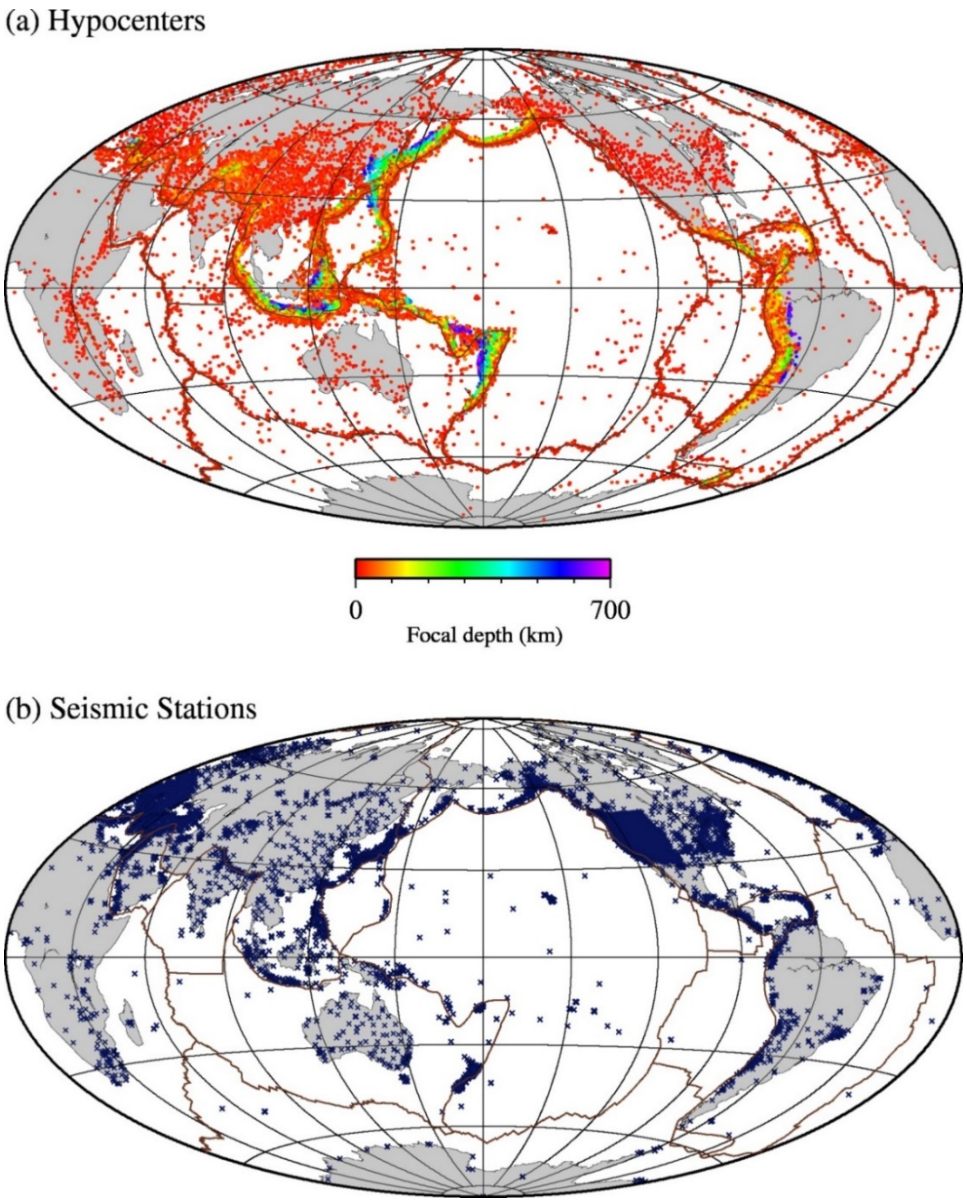

Fig. S1. Distribution of the 39,323 earthquakes **(a)** and 9141 seismic stations **(b)** used in tomographic inversions. The colors in (a) denote the focal depth whose scale is shown below (a). The brown lines denote plate boundaries. This figure was generated using the Generic Mapping Tools version 4.5.8 (http://gmt.soest.hawaii.edu).

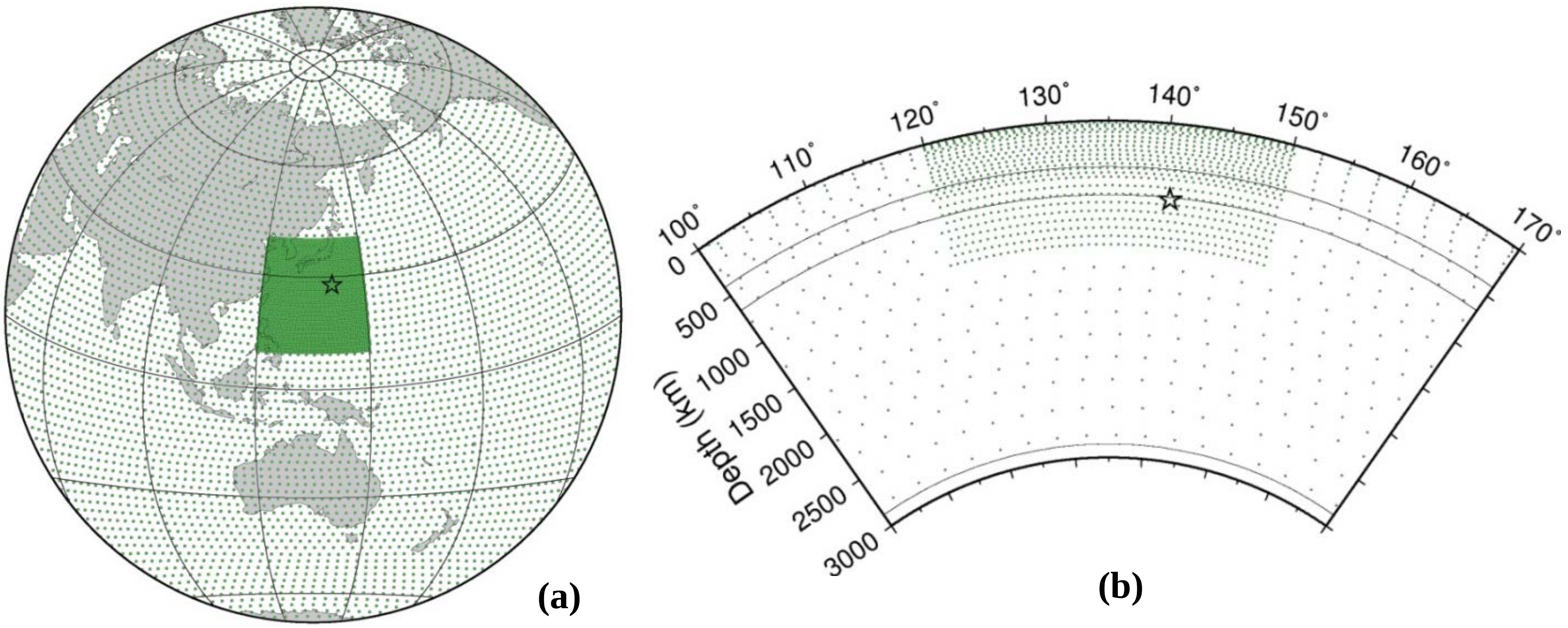

Fig. S2. Map view **(a)** and east-west vertical cross-section **(b)** showing the 3-D grid nodes adopted for the tomographic inversion. In the target area (the green box in **(a)**), a denser grid is arranged, whereas a coarser grid is set up in the surrounding crust and mantle of the Earth. The open star denotes the hypocenter of the 2015 Bonin deep earthquake. The three thin lines in (b) denote the 410 and 670 km discontinuities and the core-mantle boundary. The numbers atop (b) denote the eastern longitudes. This figure was generated using the Generic Mapping Tools version 4.5.8 (http://gmt.soest.hawaii.edu).

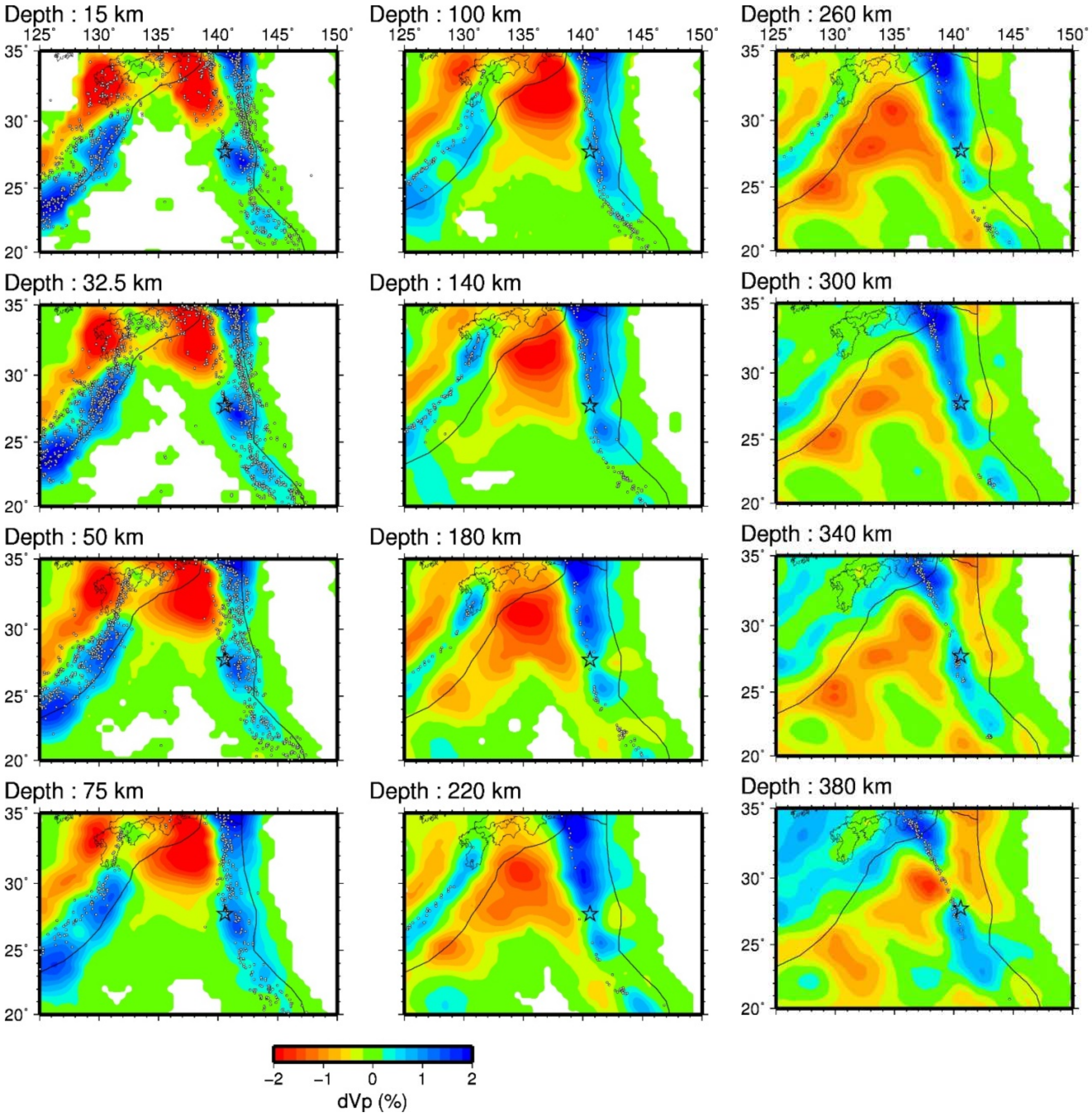

Fig. S3. Map views of P-wave tomography. The layer depth is shown atop each map. The red and blue colors denote low and high velocities, respectively, whose scale is shown at the bottom. The open star denotes the epicenter of the 2015 Bonin deep earthquake. The black lines denote plate boundaries. The white dots denote seismicity within a 30-km depth of each layer. This figure was generated using the Generic Mapping Tools version 4.5.8 (http://gmt.soest.hawaii.edu).

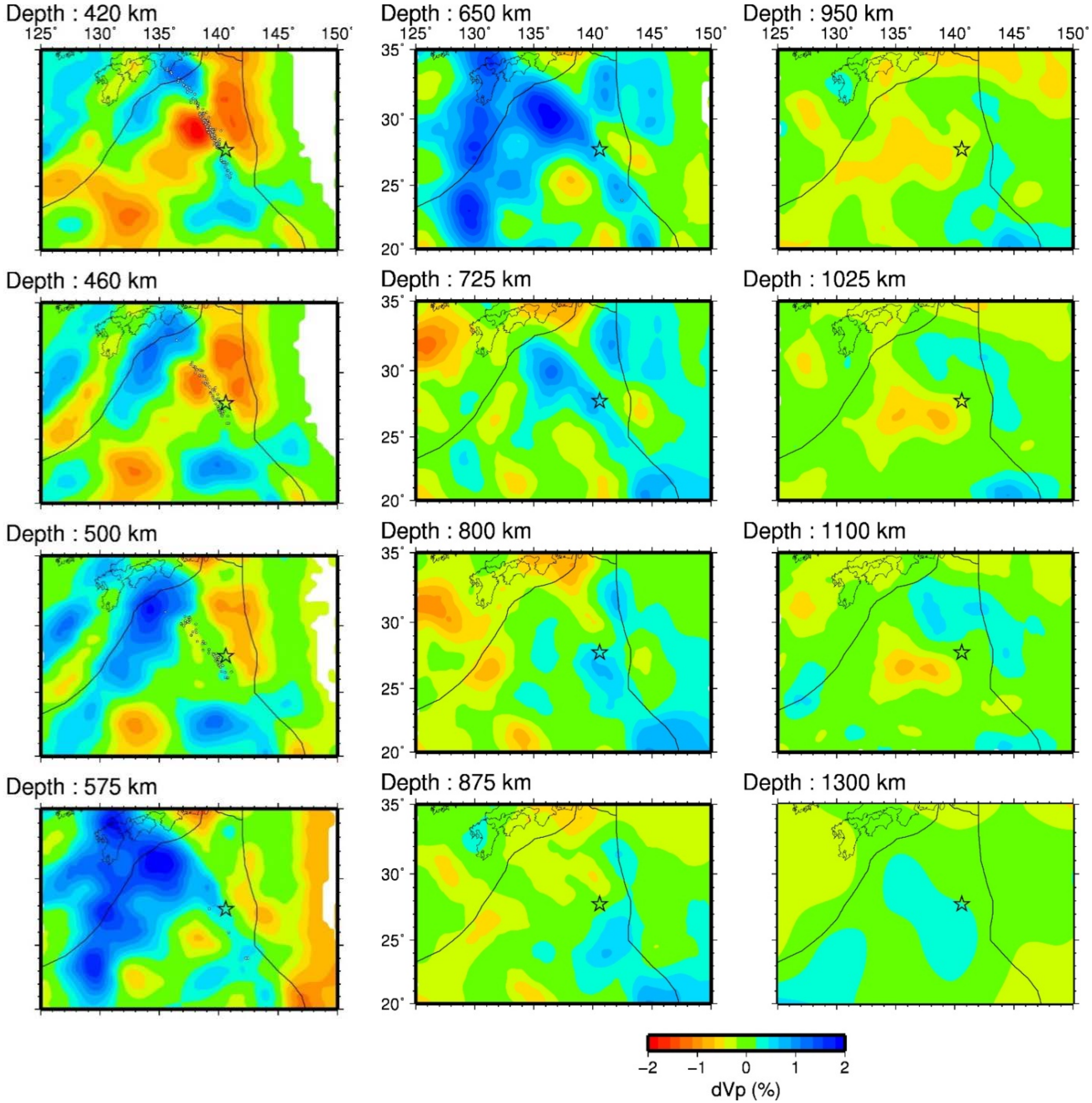

Fig. S3 (continued).

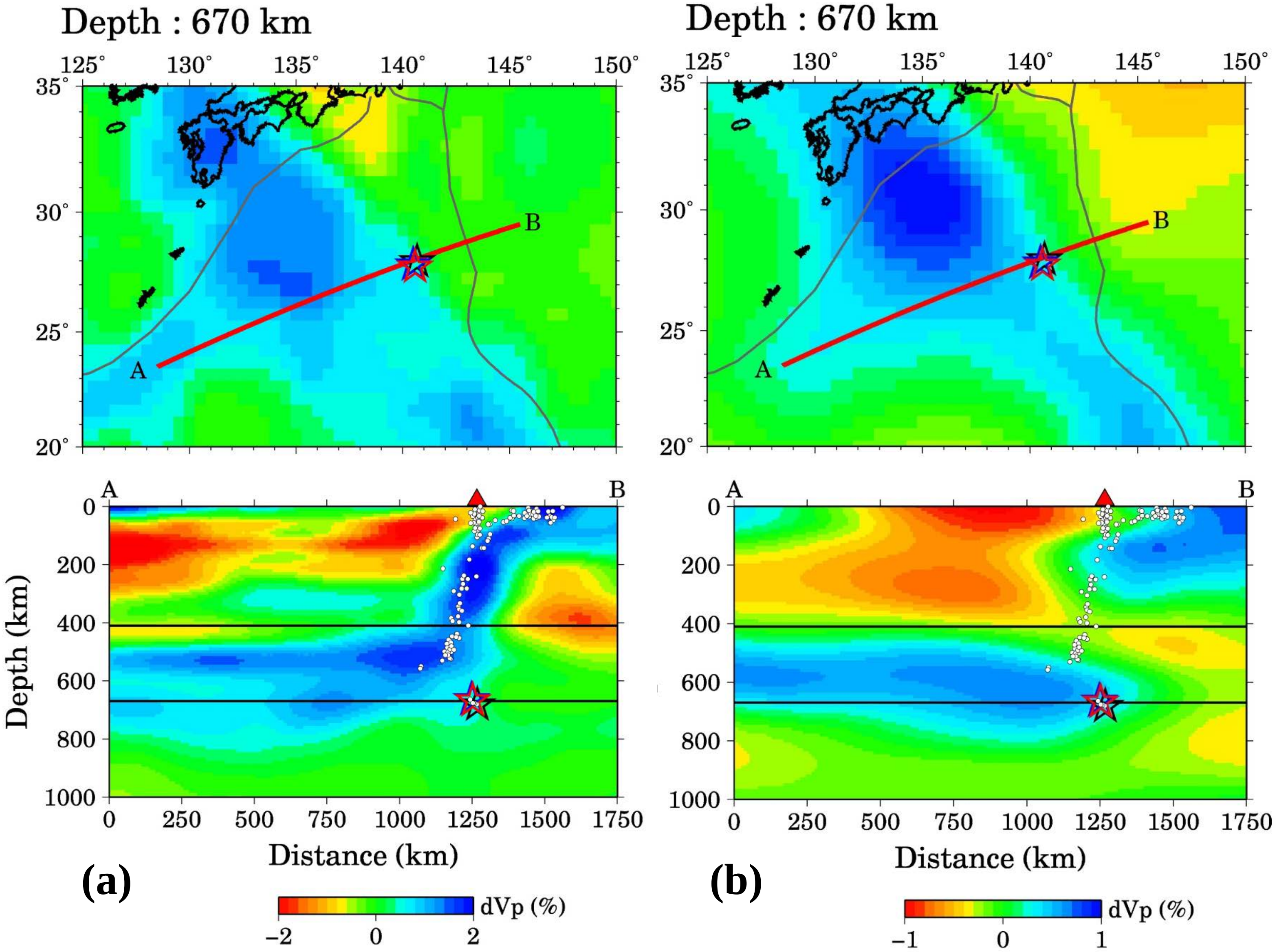

Fig. S4. The same as Fig. 5 but determined by (a) *Obayashi et al*. (2013) and (b) *Zhao et al*. (2013). The black, blue and red starts denote the hypocentral locations of the 2015 Bonin deep earthquake (Mw 7.9) determined the JMA, USGS and this study, respectively (see also Table S1). This figure was generated using the Generic Mapping Tools version 4.5.8 (http://gmt.soest.hawaii.edu).

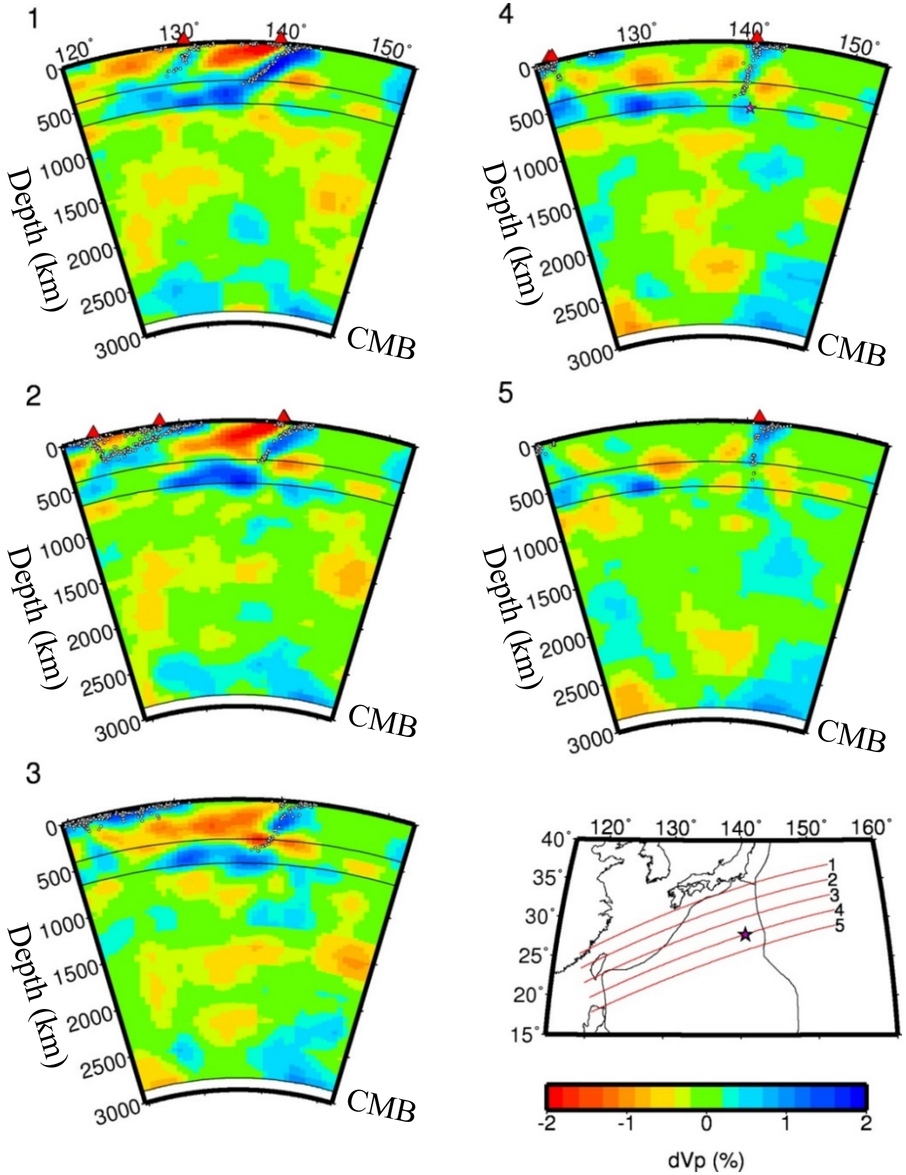

Fig. S5. Vertical cross-sections of P-wave tomography down to the core-mantle boundary (CMB) along the profiles showing in the inset map. The red and blue colors denote low and high velocities, respectively, whose scale is shown at the bottom. This figure was generated using the Generic Mapping Tools version 4.5.8 (http://gmt.soest.hawaii.edu).

A checkerboard resolution test with a lateral grid interval of 1°

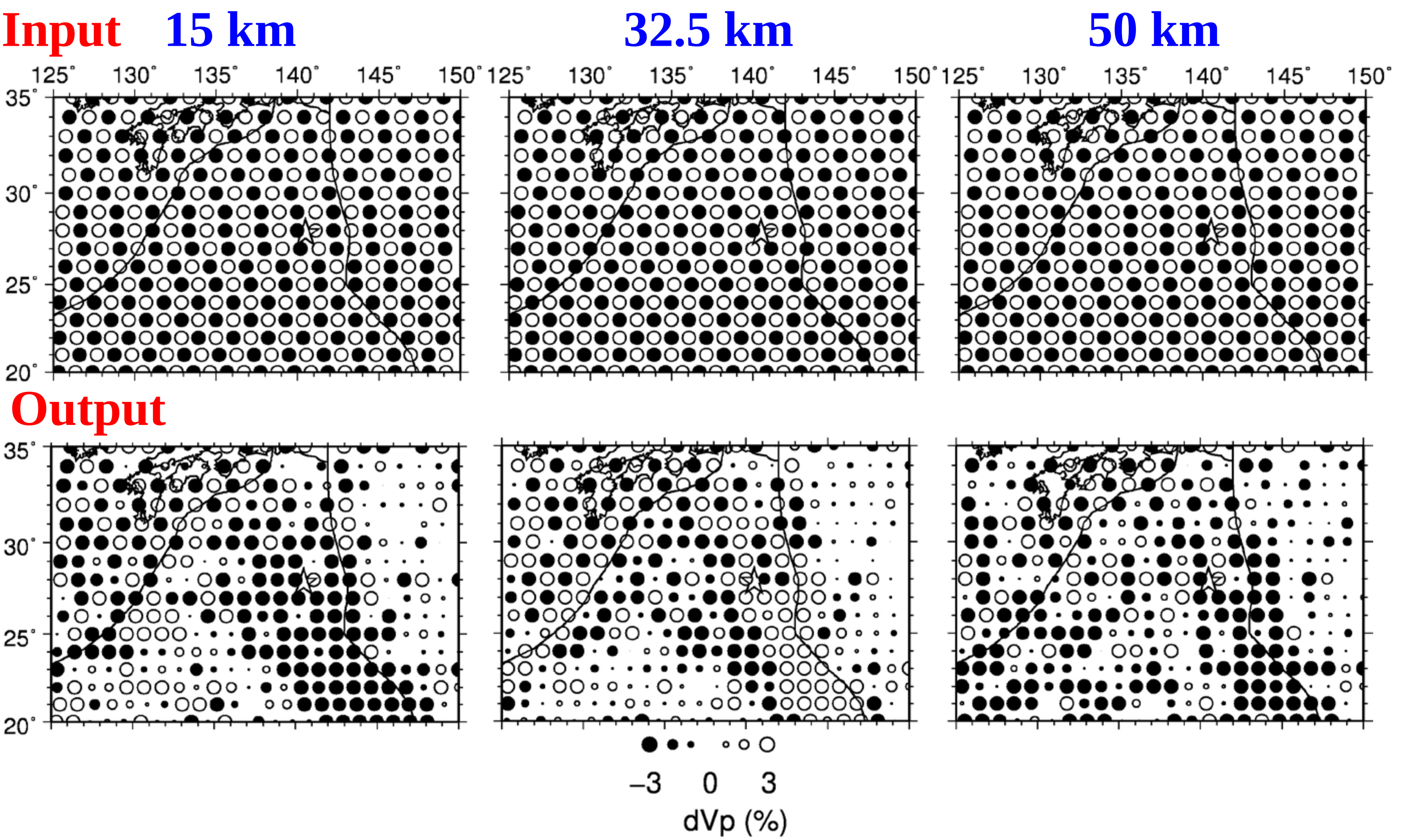

Fig. S6. Map views showing the input model (upper panels) and output results (lower panels) of a checkerboard resolution test. The open and solid circles denote high and low velocities, respectively, whose scale is shown at the bottom. The black lines denote plate boundaries. The depth of each layer is shown above the upper panels. This figure was generated using the Generic Mapping Tools version 4.5.8 (http://gmt.soest.hawaii.edu).

A checkerboard resolution test with a lateral grid interval of 1°

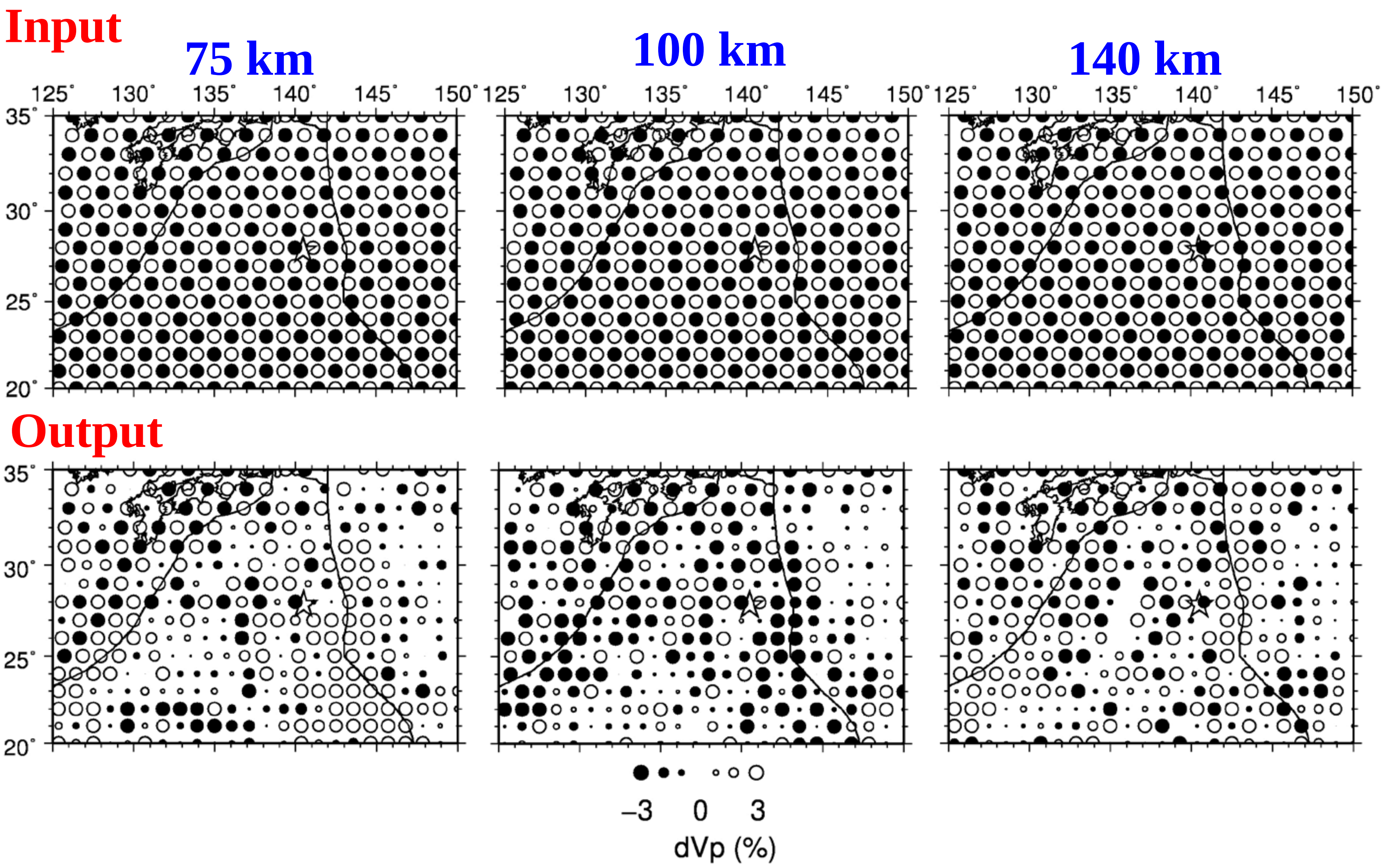

Fig. S6 (continued).

A checkerboard resolution test with a lateral grid interval of 1°

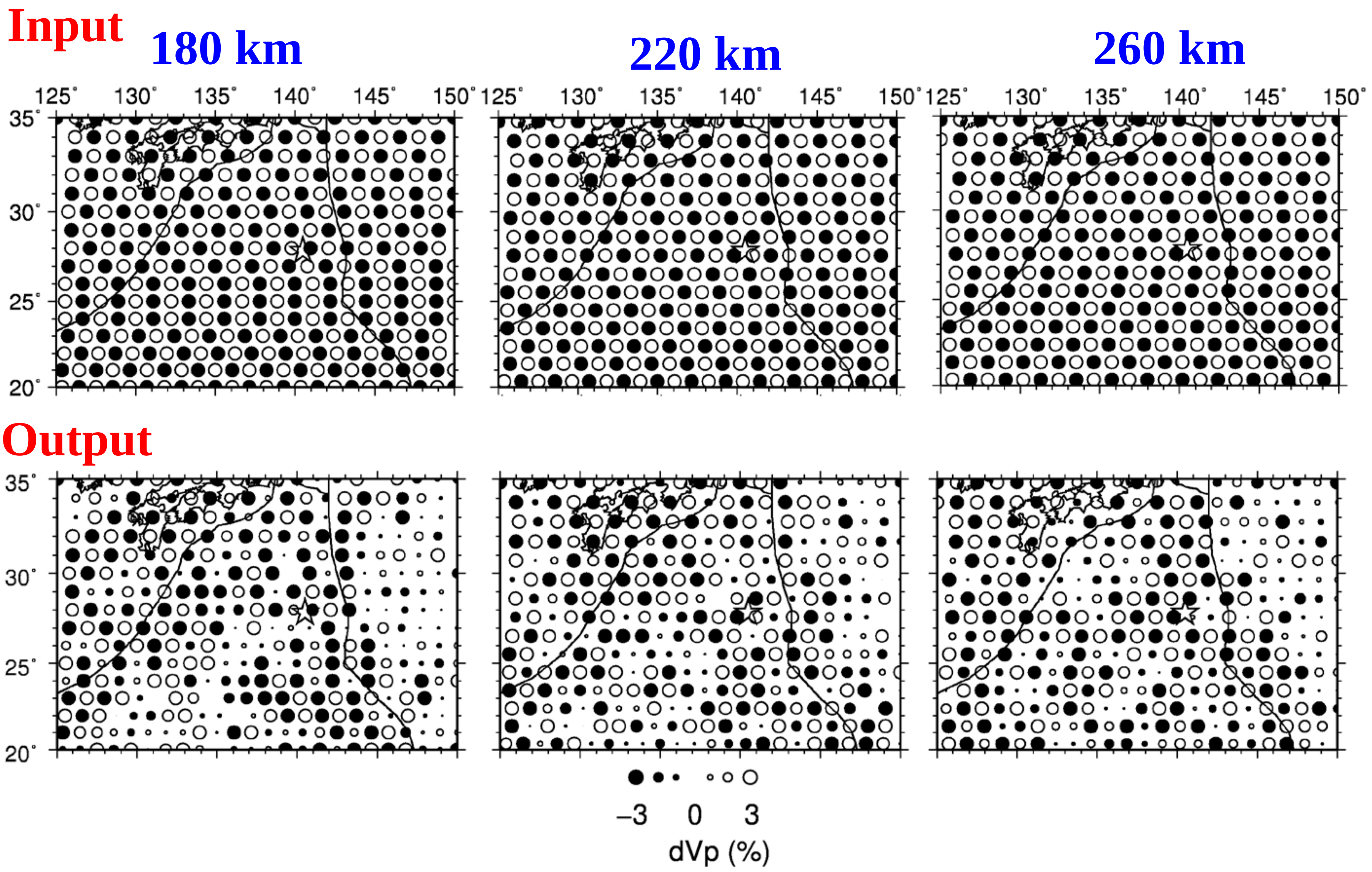

Fig. S6 (continued).

A checkerboard resolution test with a lateral grid interval of 1°

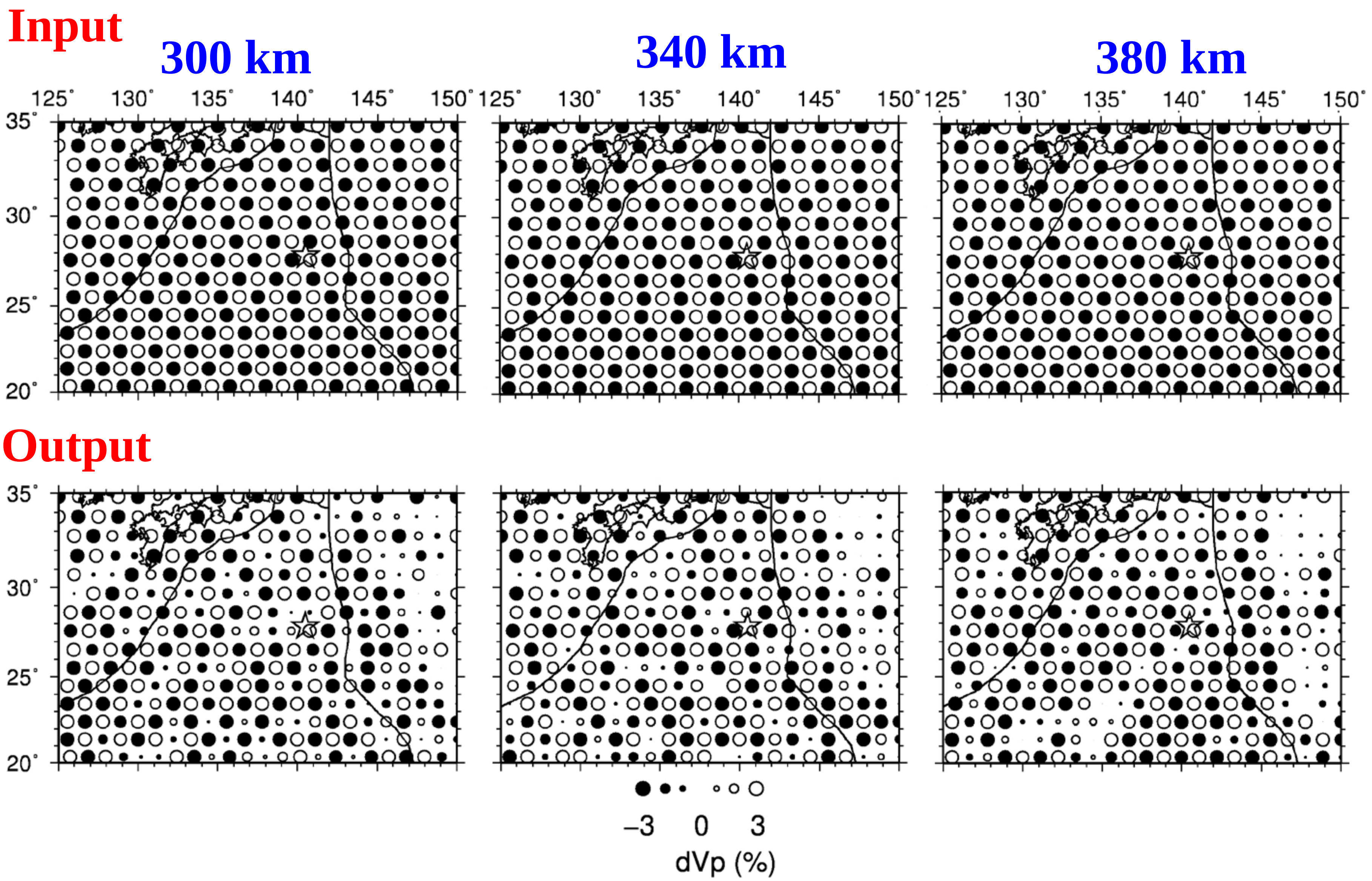

Fig. S6 (continued).

A checkerboard resolution test with a lateral grid interval of 1°

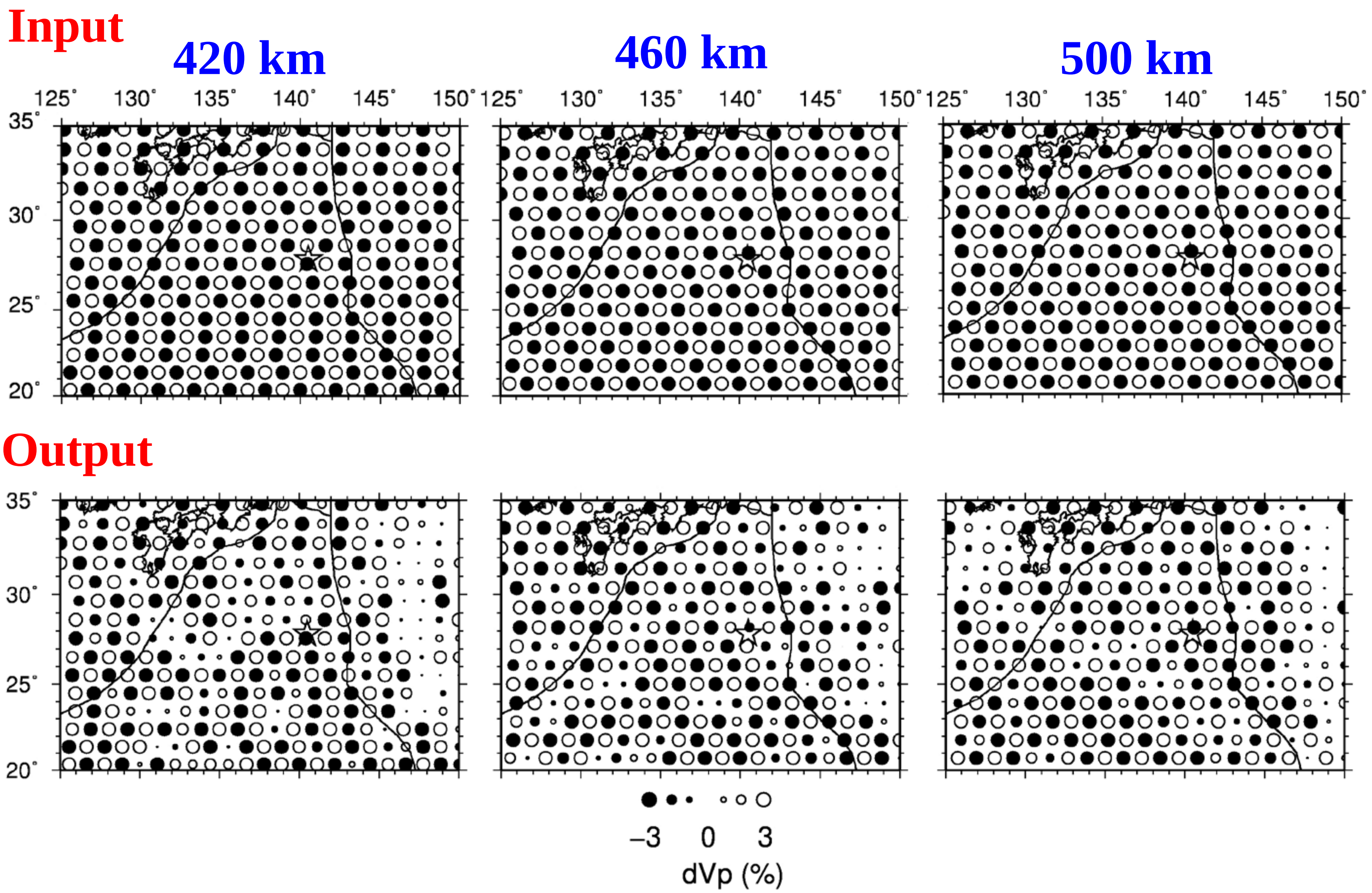

Fig. S6 (continued).

A checkerboard resolution test with a lateral grid interval of 1°

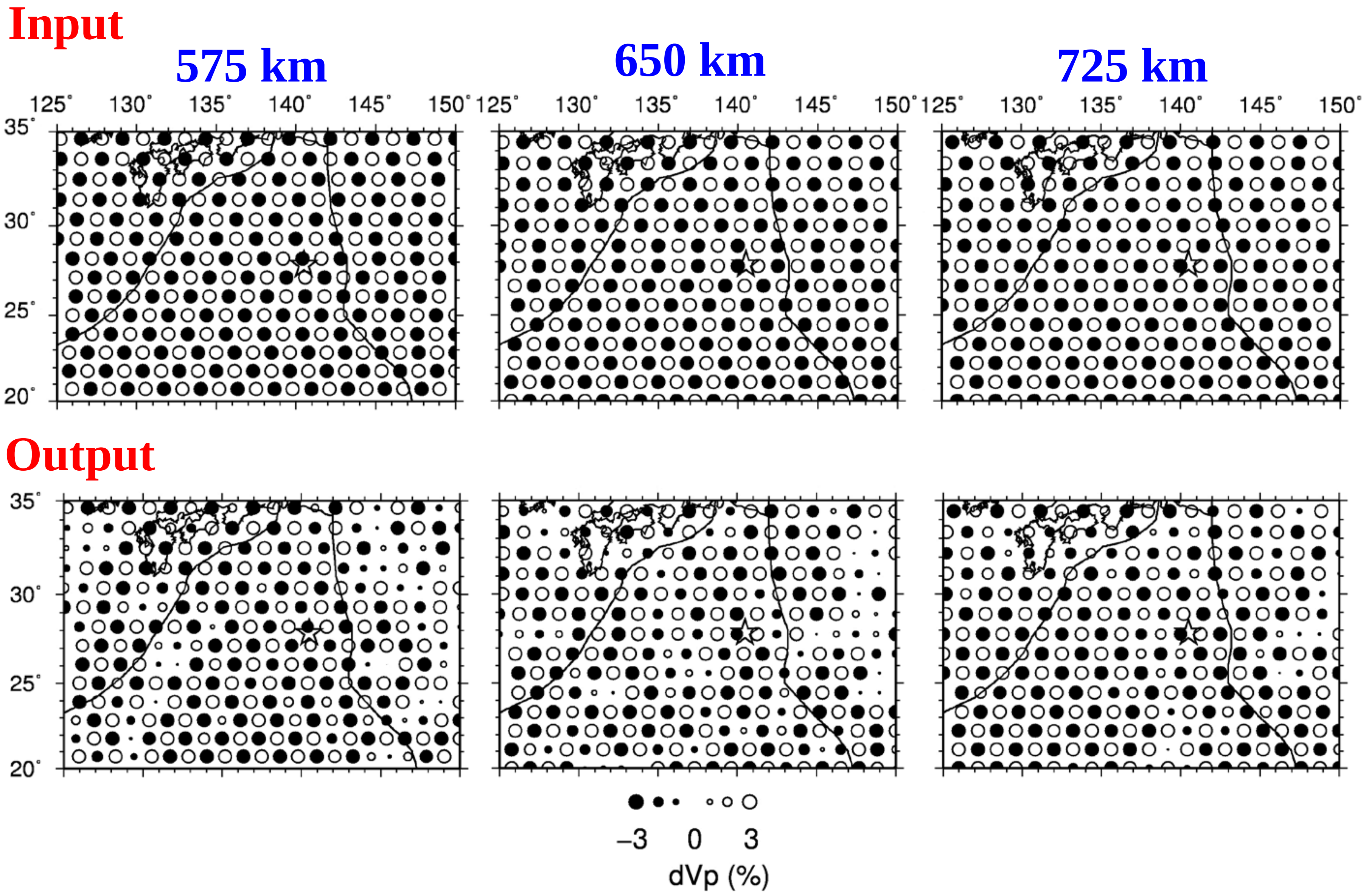

Fig. S6 (continued).

A checkerboard resolution test with a lateral grid interval of 1°

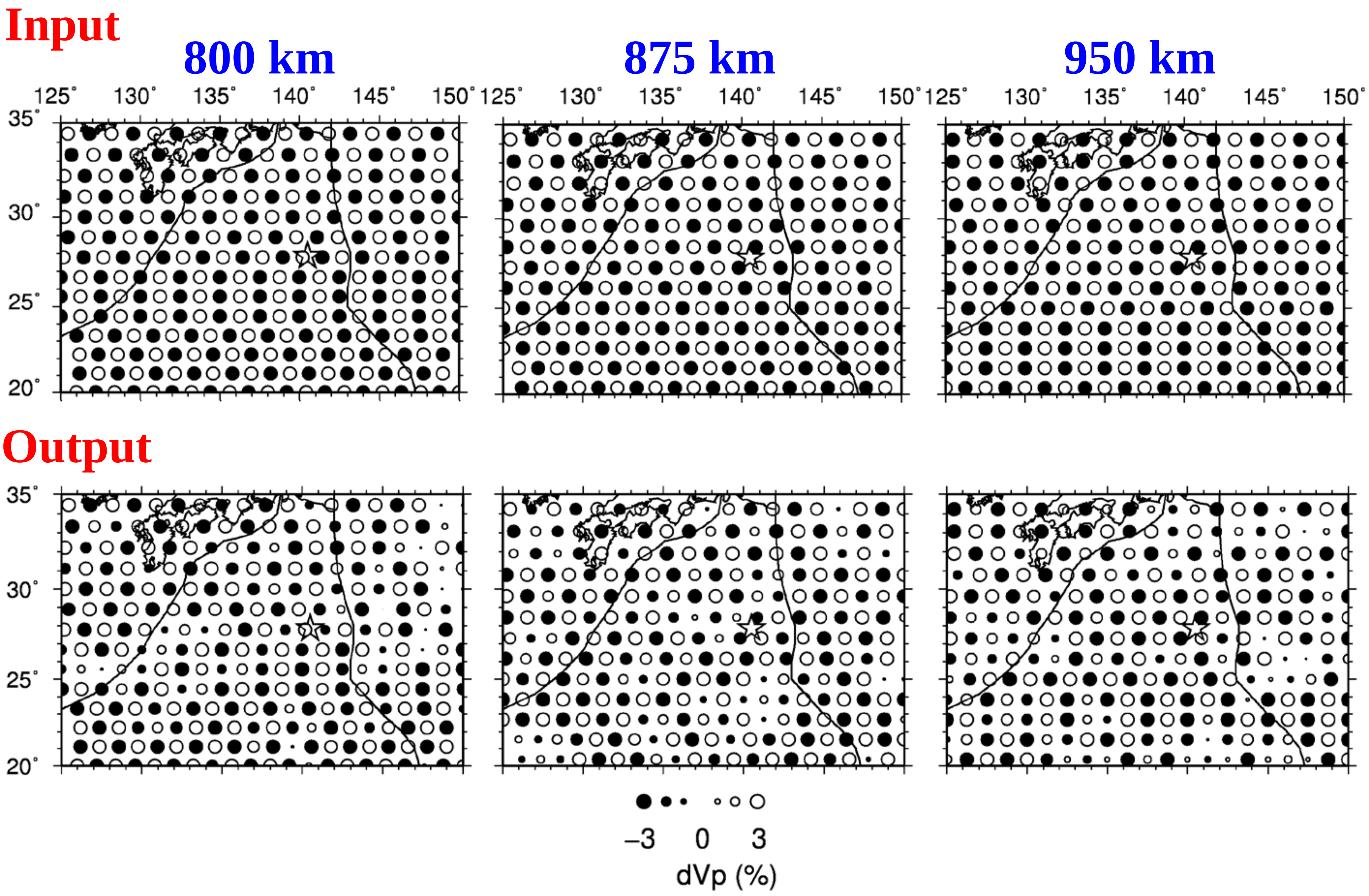

Fig. S6 (continued).

Synthetic resolution test

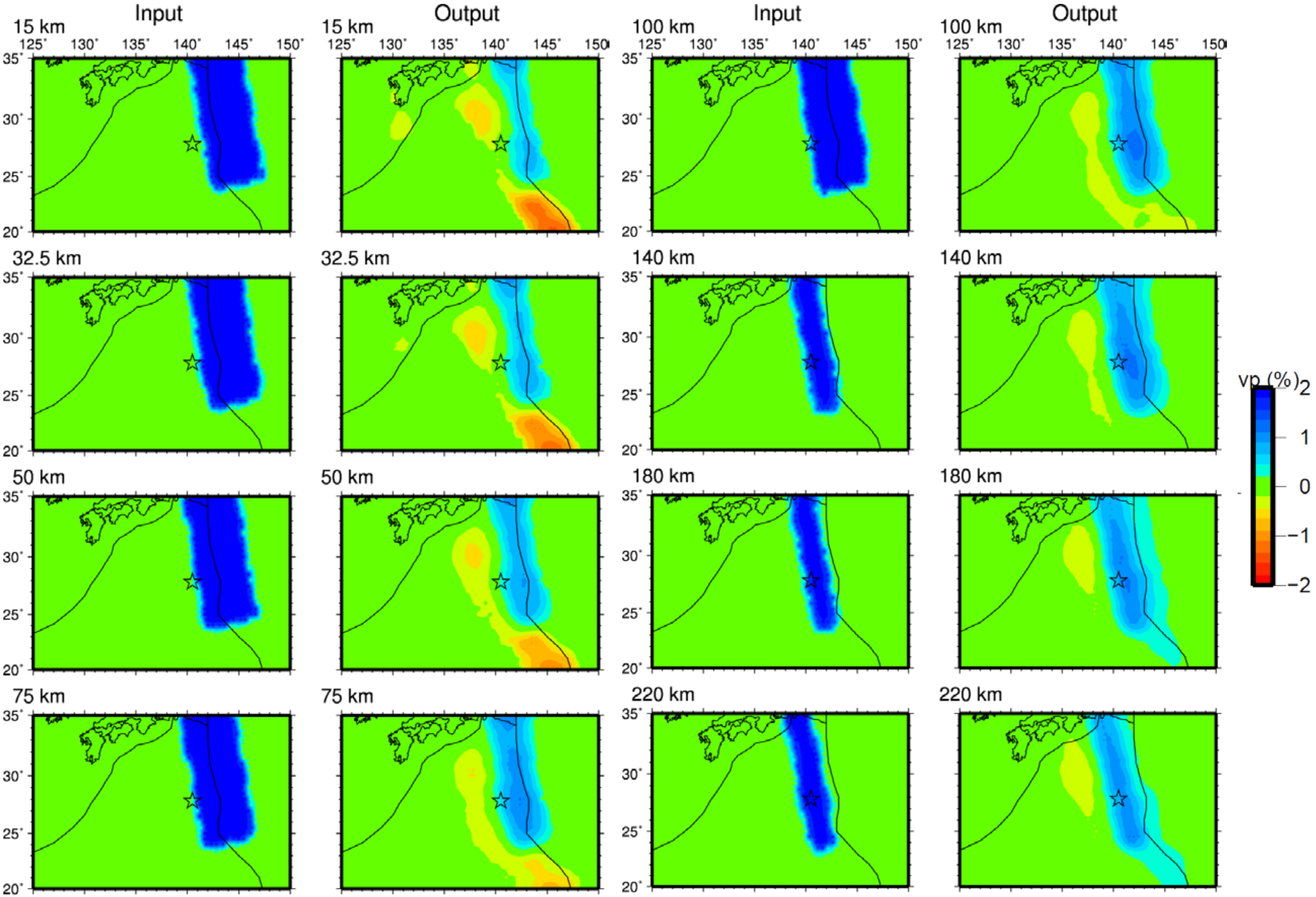

Fig. S7. Map views showing the input model and output results of a synthetic resolution test. The blue and red colors denote high and low velocities, respectively, whose scale is shown on the right. The black lines denote plate boundaries. The layer depth is shown above each map. This figure was generated using the Generic Mapping Tools version 4.5.8 (http://gmt.soest.hawaii.edu).

Synthetic resolution test

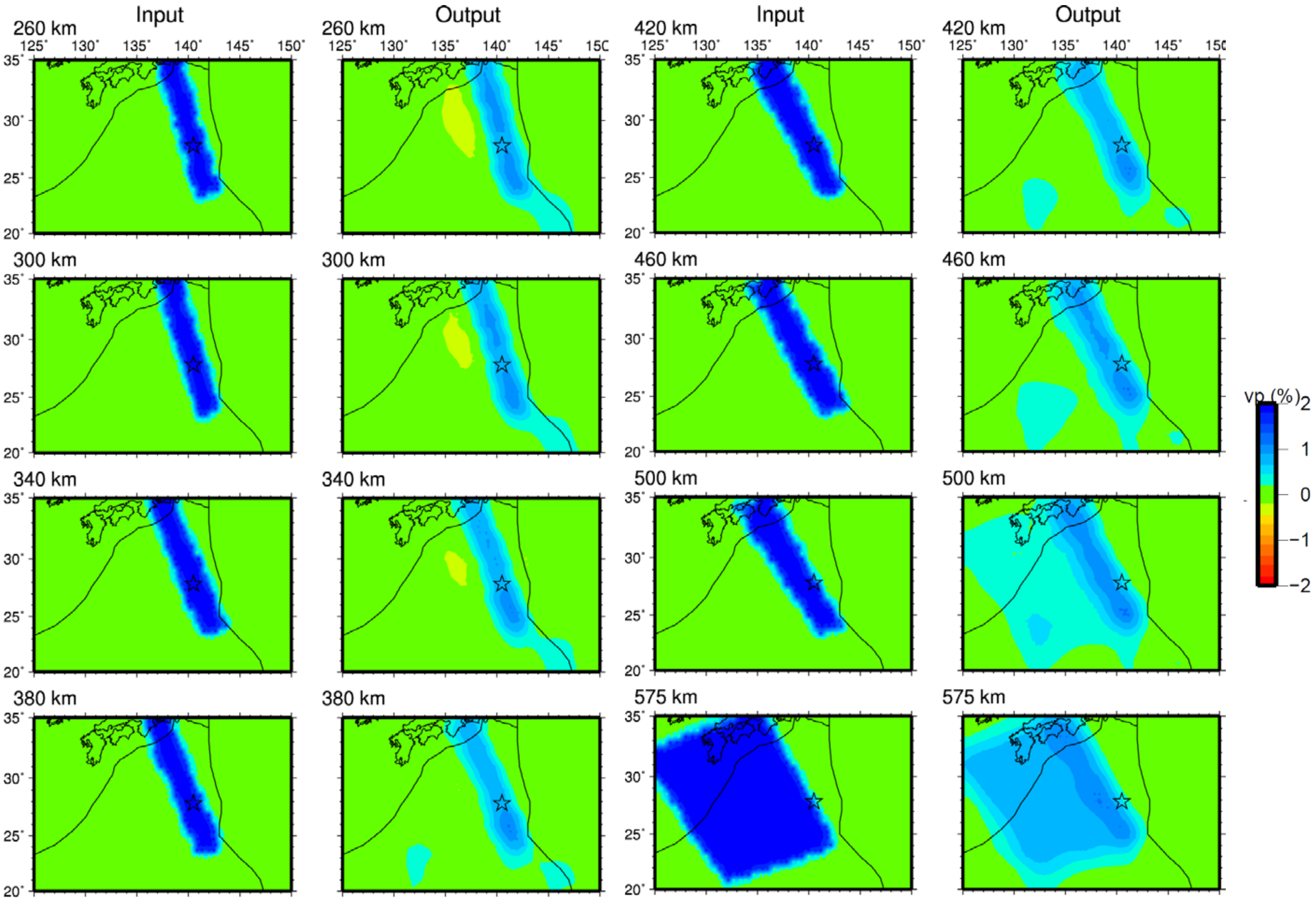

Fig. S7 (continued).

Synthetic resolution test

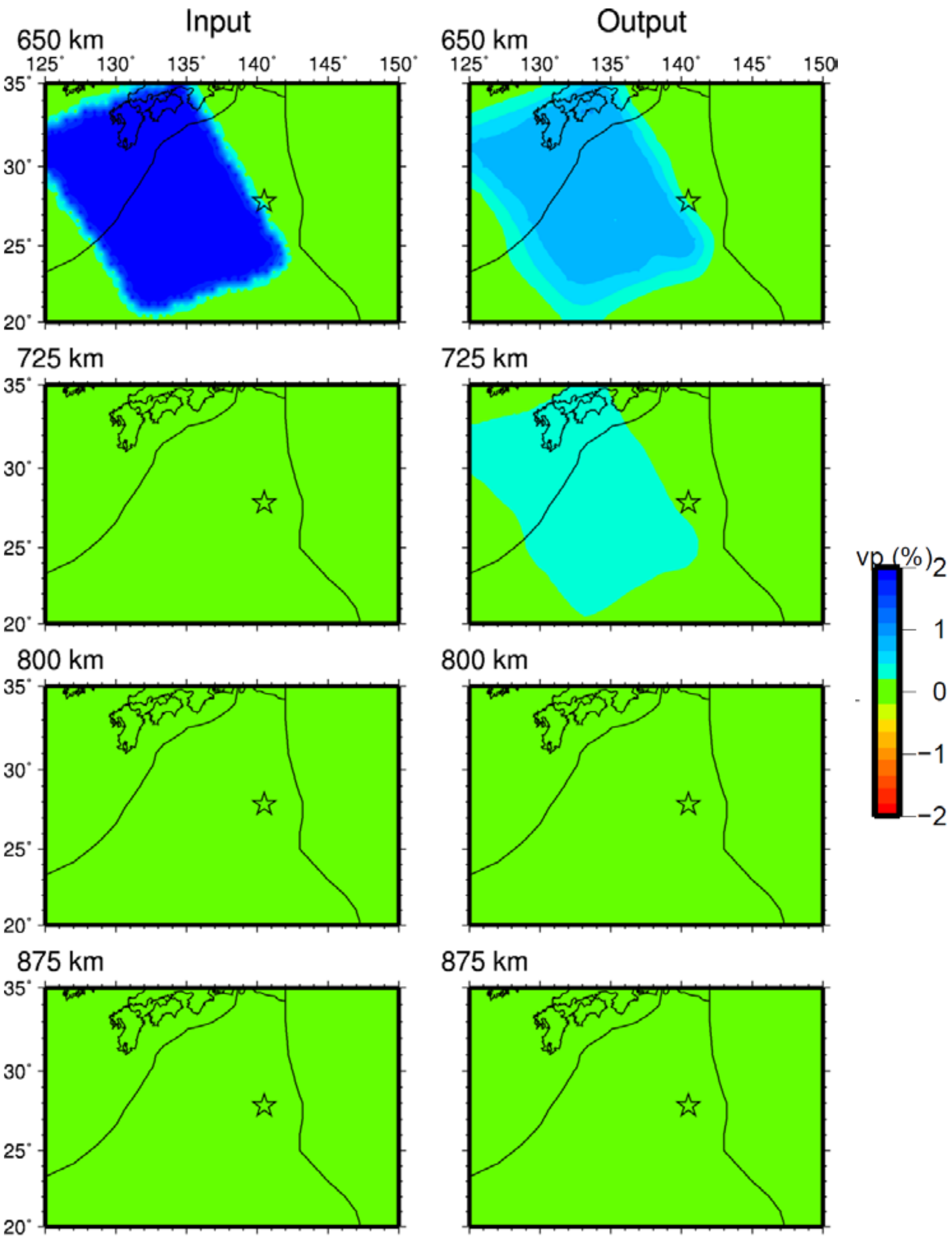

Fig. S7 (continued).

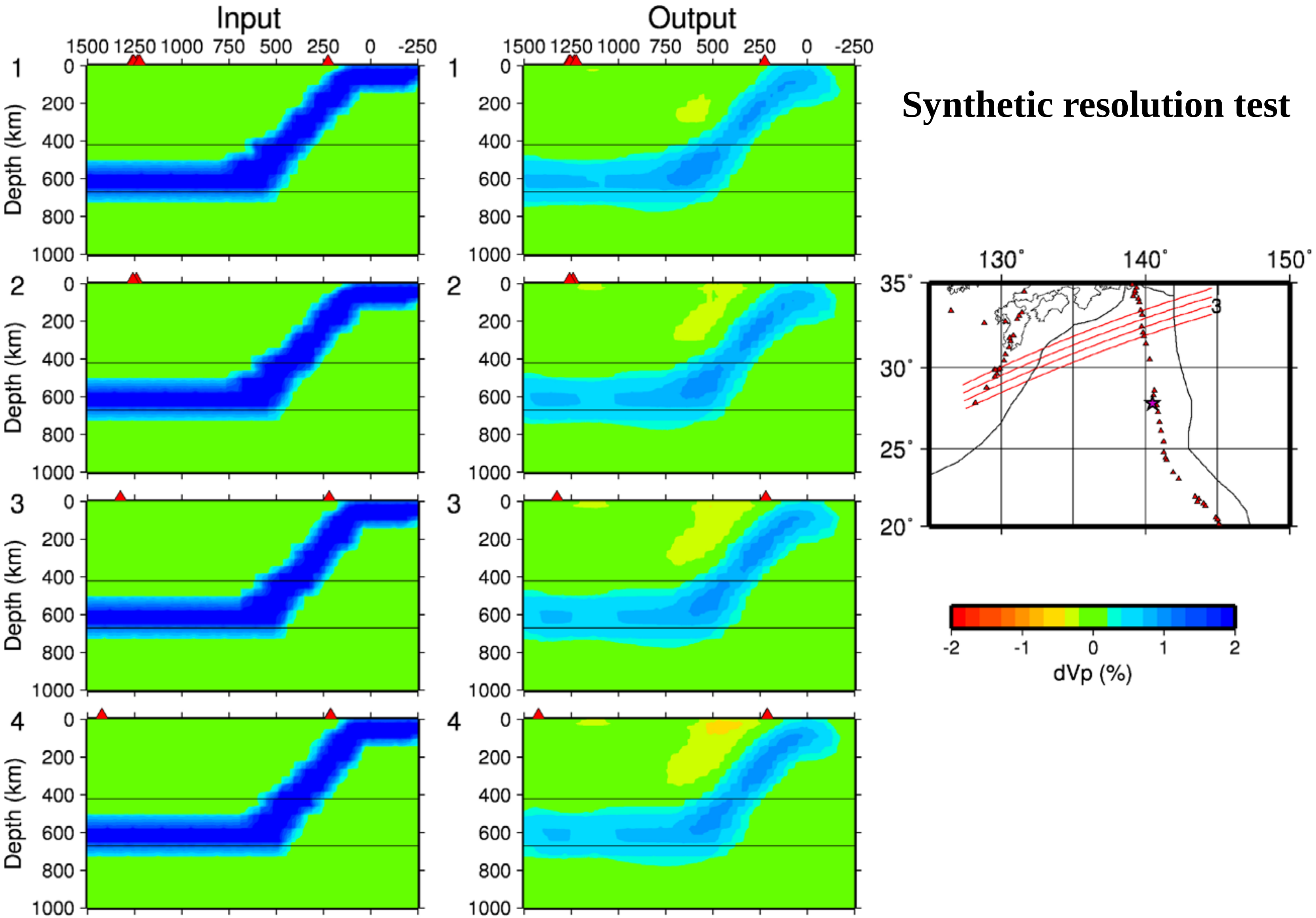

Fig. S8. Vertical cross-sections showing the input model (left) and output results (right) of a synthetic resolution test. Locations of the cross-sections are shown in red lines on the inset map. The blue and red colors denote high and low velocities, respectively, whose scale is shown below the inset map. The two black lines in each cross-section denote the 410 and 670 km discontinuities. The red triangles denote active volcanoes. This figure was generated using the Generic Mapping Tools version 4.5.8 (http://gmt.soest.hawaii.edu).

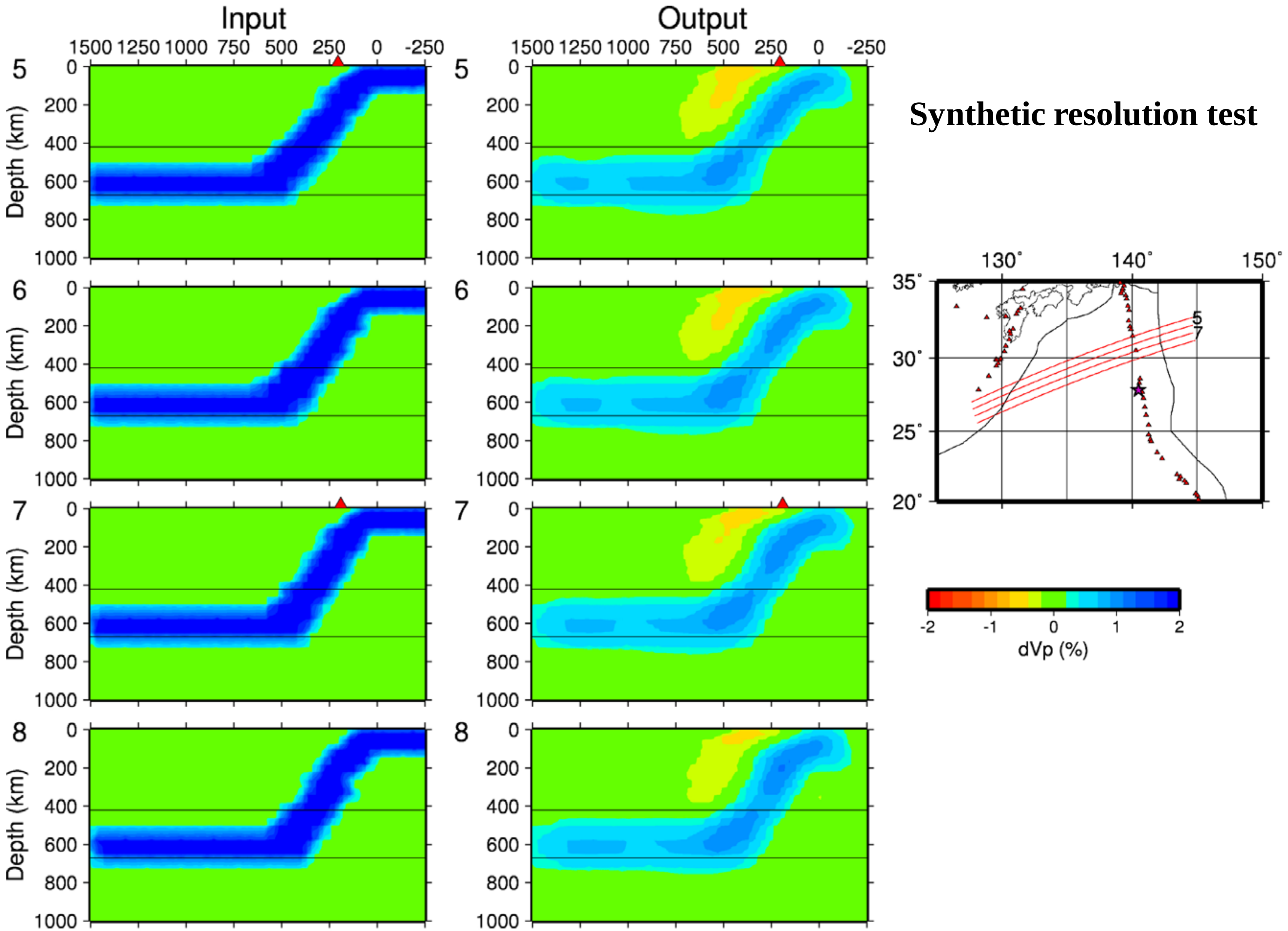

Fig. S8 (continued).

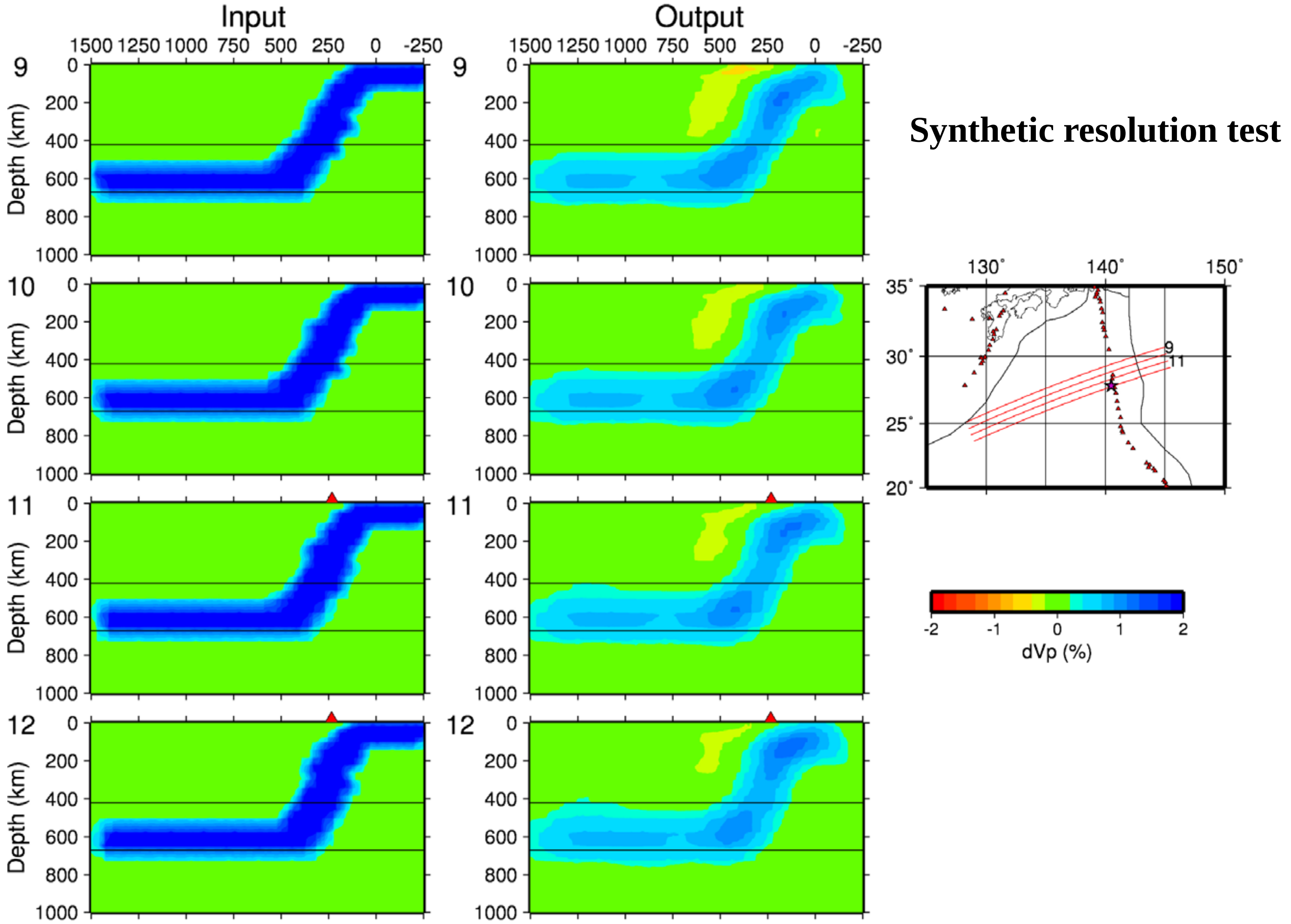

Fig. S8 (continued).

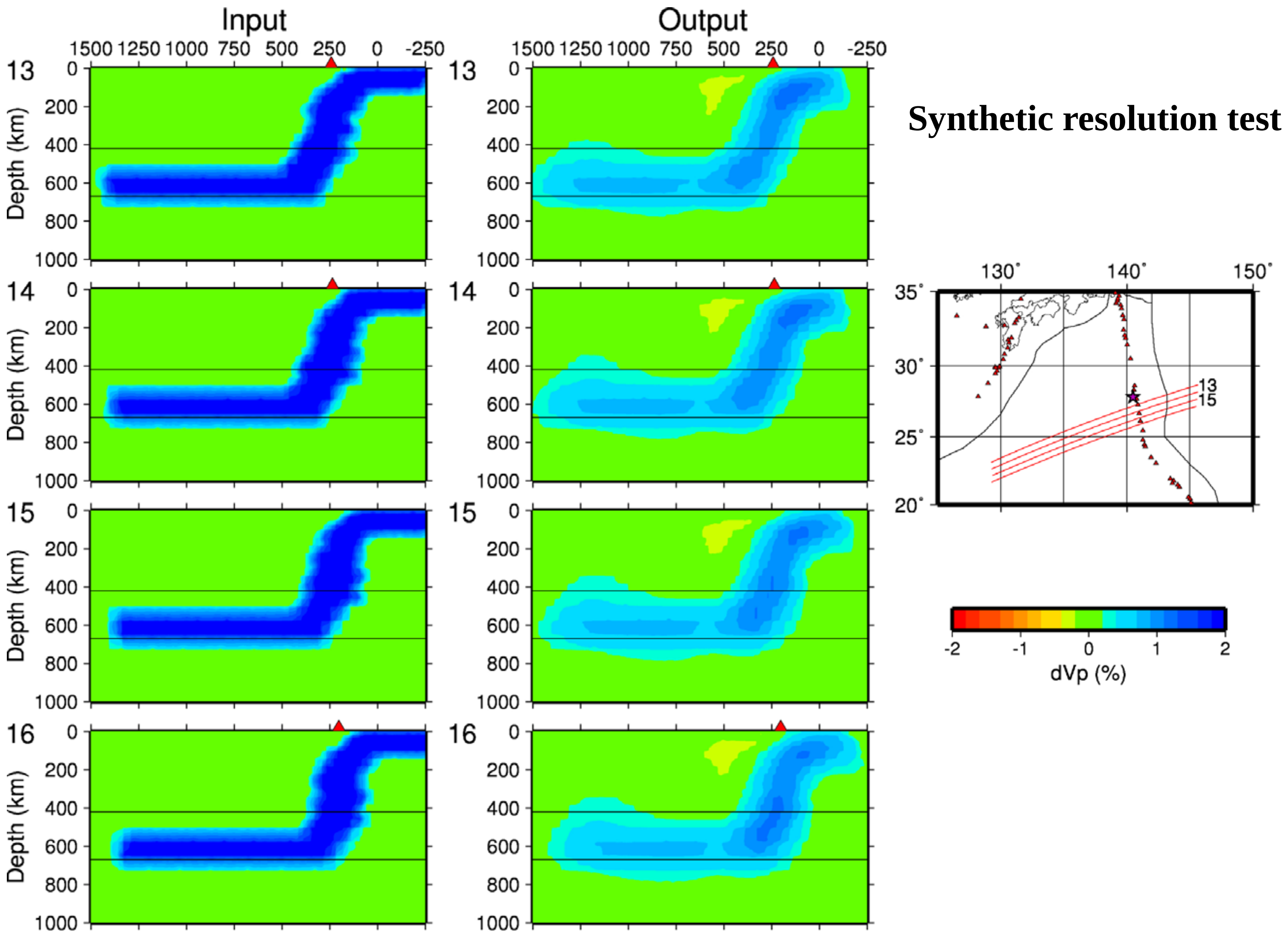

Fig. S8 (continued).

Synthetic resolution test

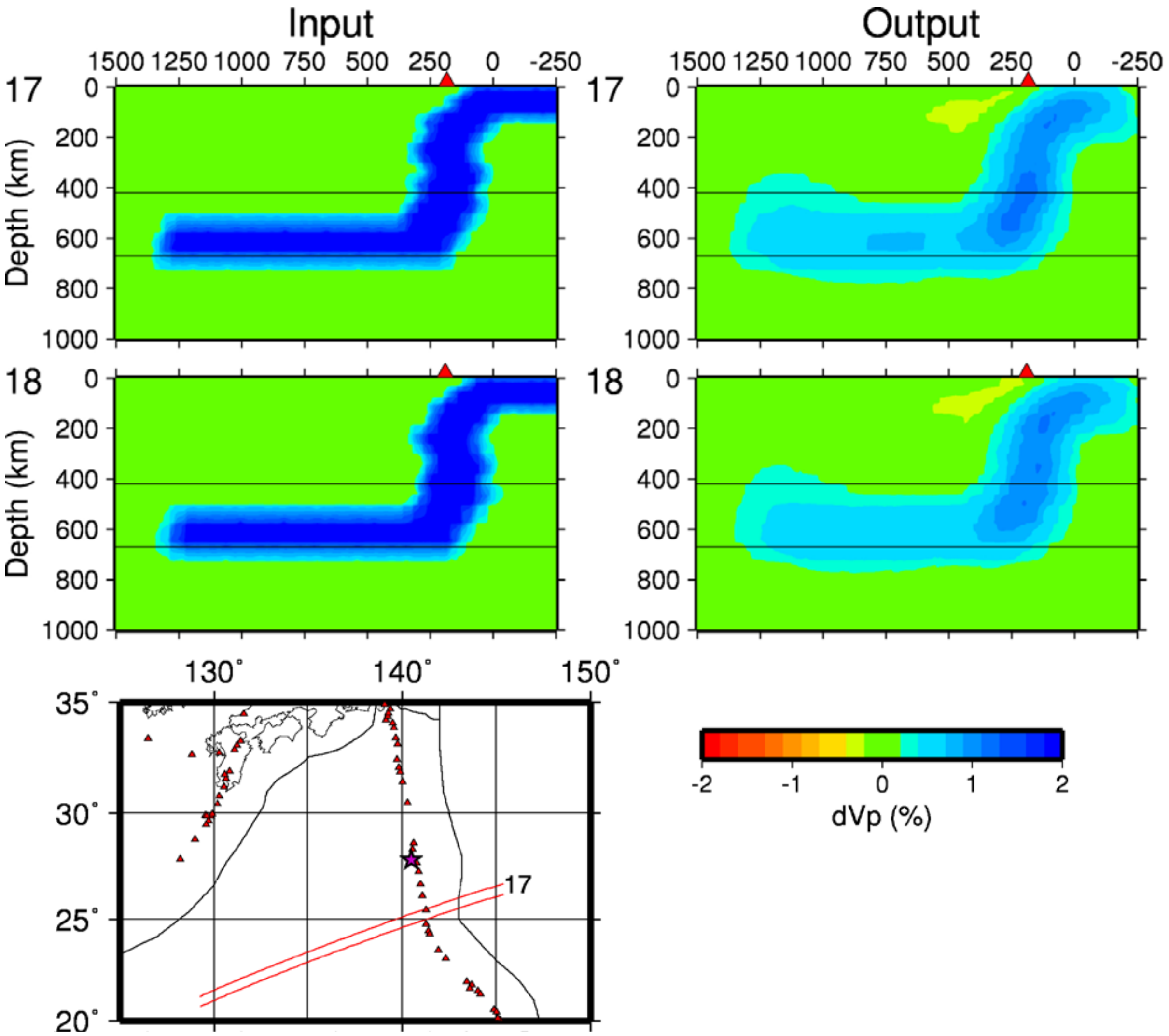

Fig. S8 (continued).

Restoring resolution test

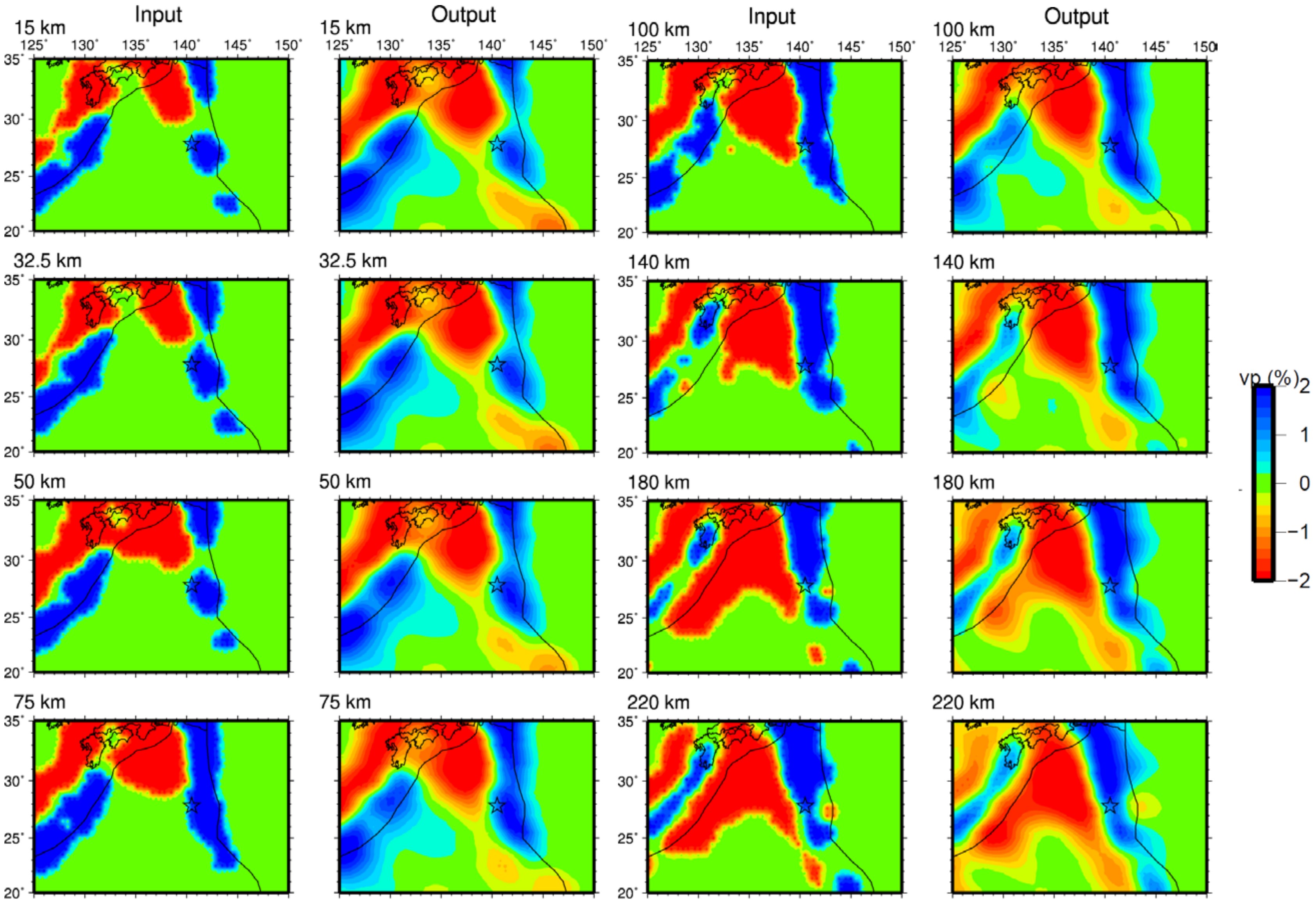

Fig. S9. Map views showing the input model and output results of a restoring resolution test. The blue and red colors denote high and low velocities, respectively, whose scale is shown on the right. The black lines denote plate boundaries. The depth of each layer is shown above each map. This figure was generated using the Generic Mapping Tools version 4.5.8 (http://gmt.soest.hawaii.edu).

Restoring resolution test

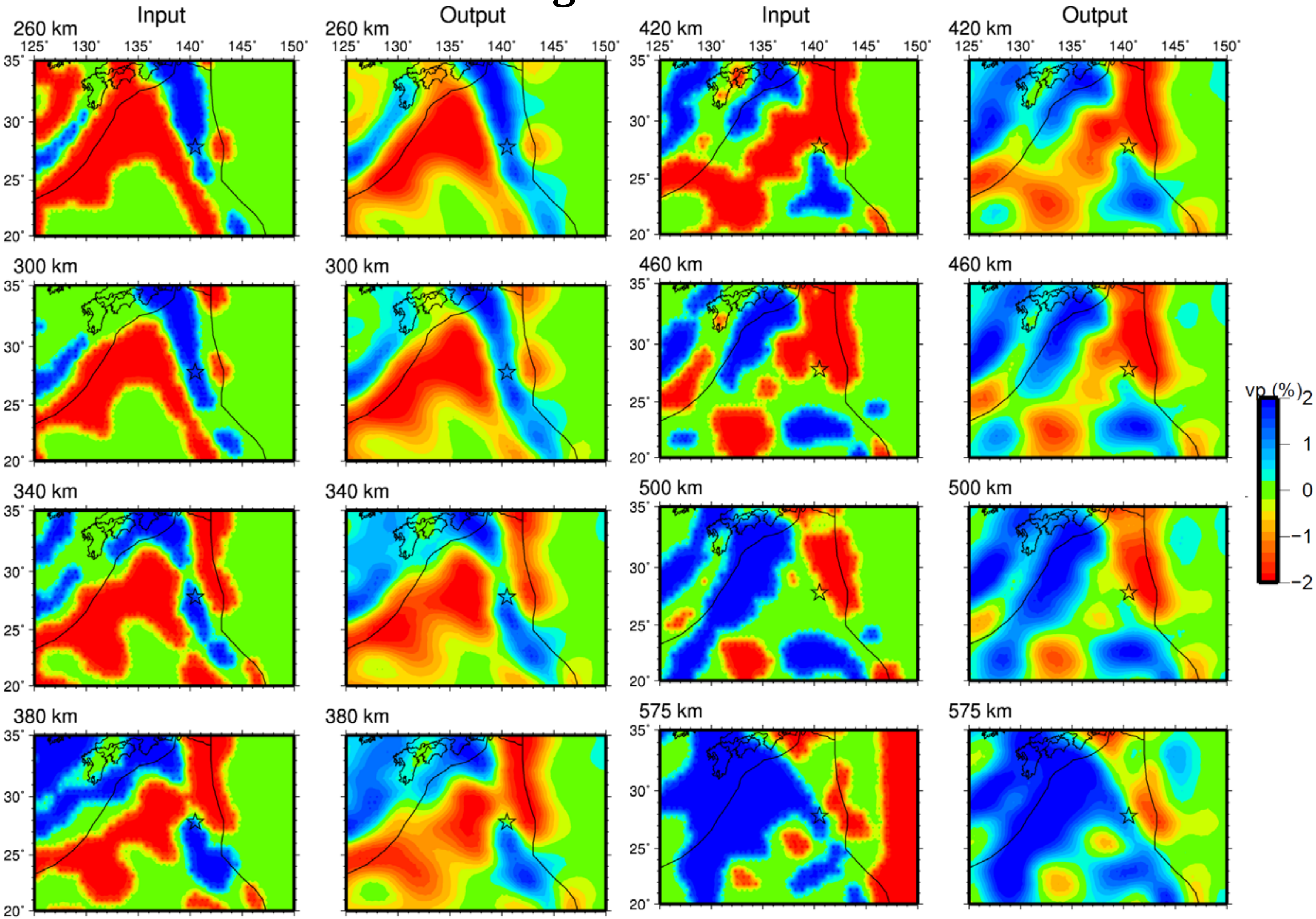

Fig. S9 (continued).

Restoring resolution test

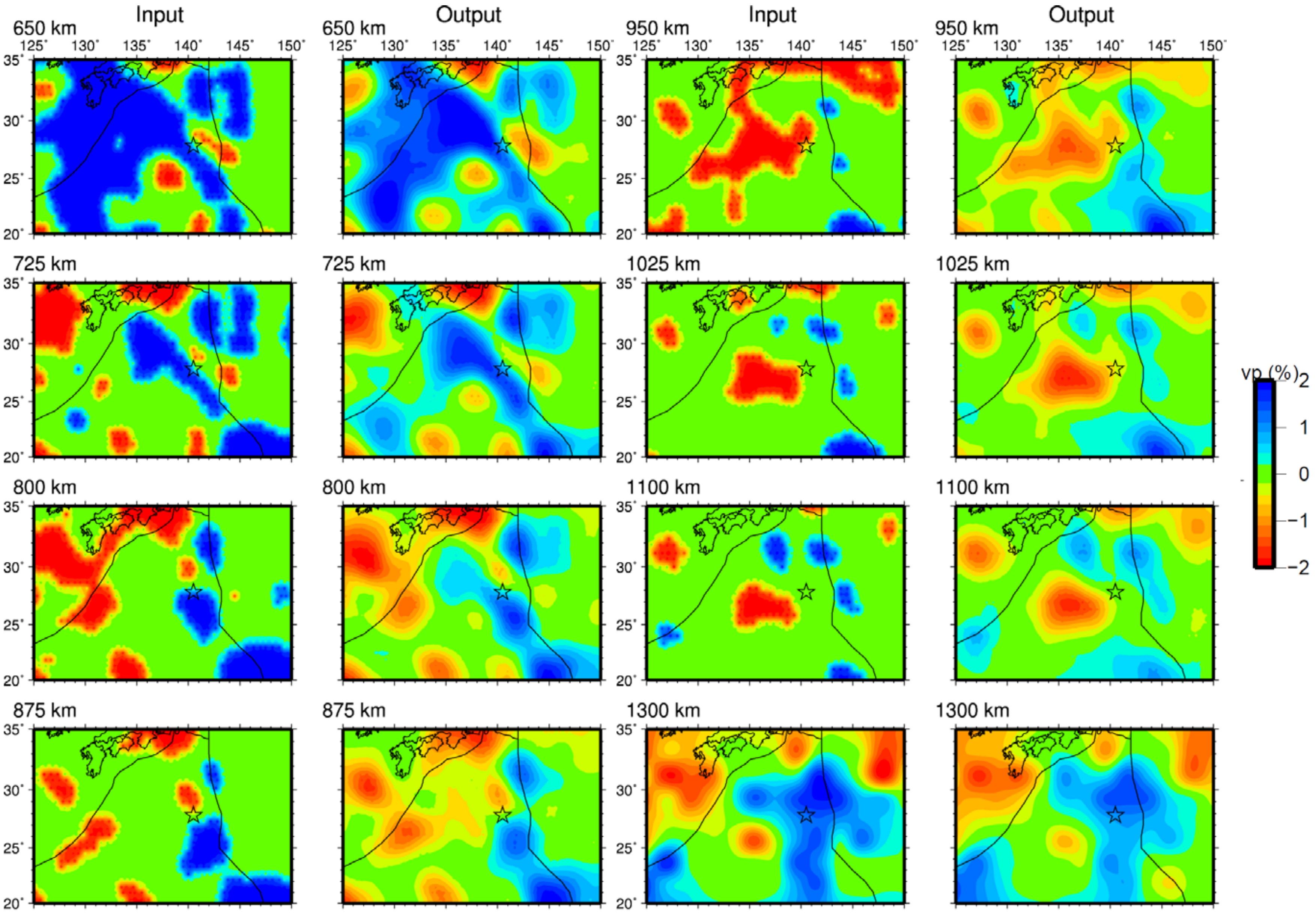

Fig. S9 (continued).

Restoring resolution test

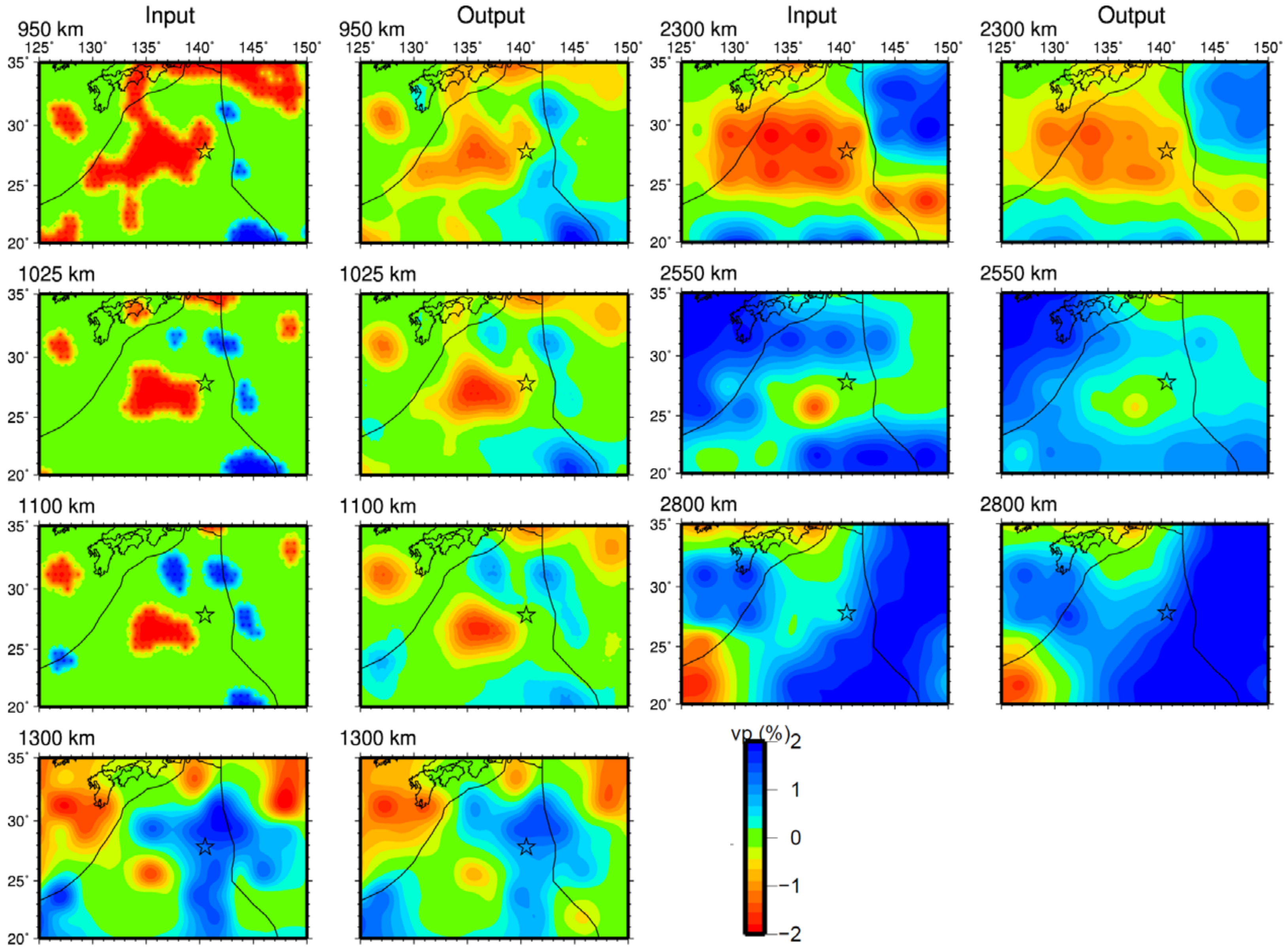

Fig. S9 (continued).

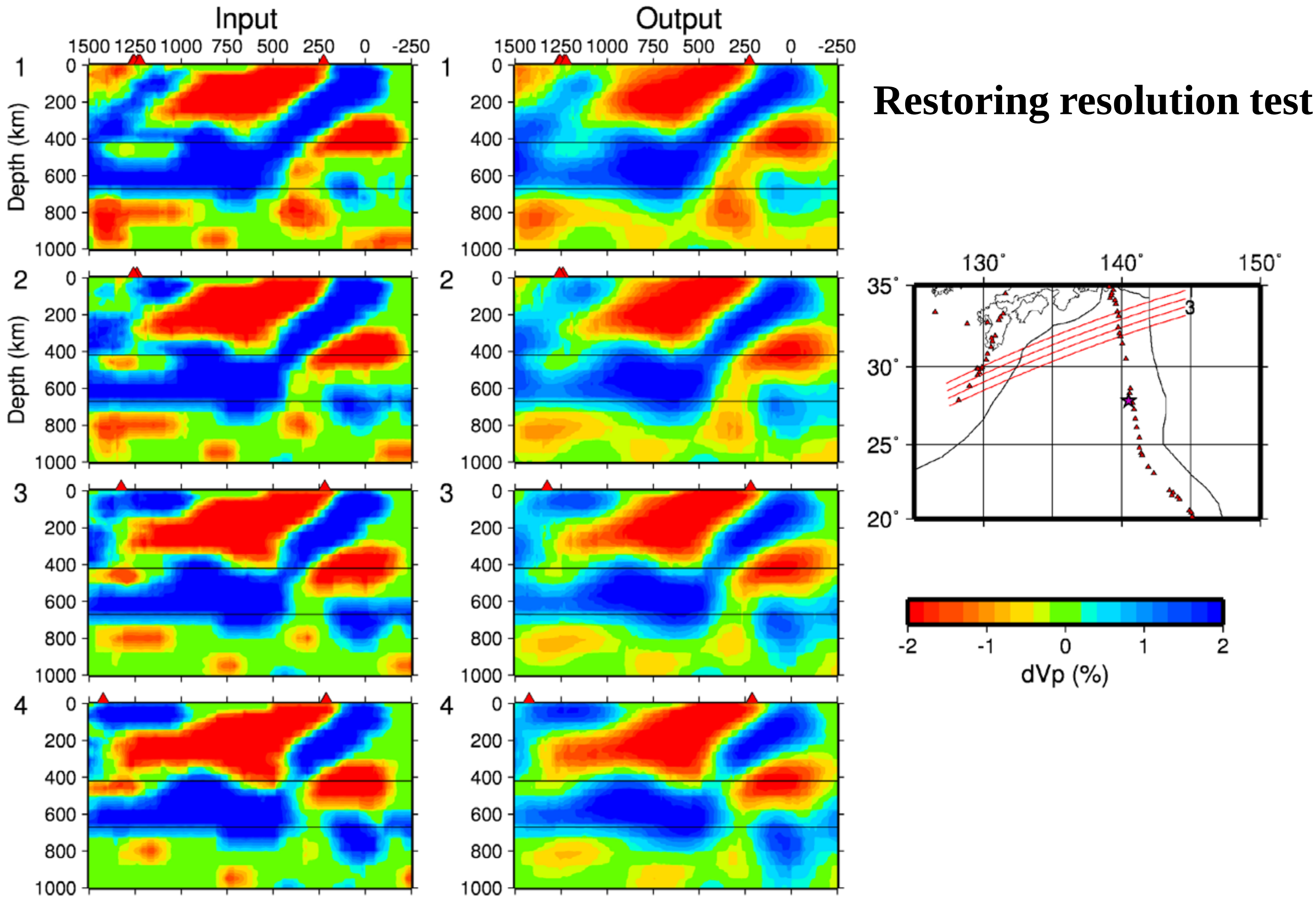

Fig. S10. Vertical cross-sections showing the input model (left) and output results (right) of a restoring resolution test. Locations of the cross-sections are shown in red lines on the inset map. The blue and red colors denote high and low velocities, respectively, whose scale is shown below the inset map. The two black lines in each cross-section denote the 410 and 670 km discontinuities. The red triangles denote active volcanoes. This figure was generated using the Generic Mapping Tools version 4.5.8 (http://gmt.soest.hawaii.edu).

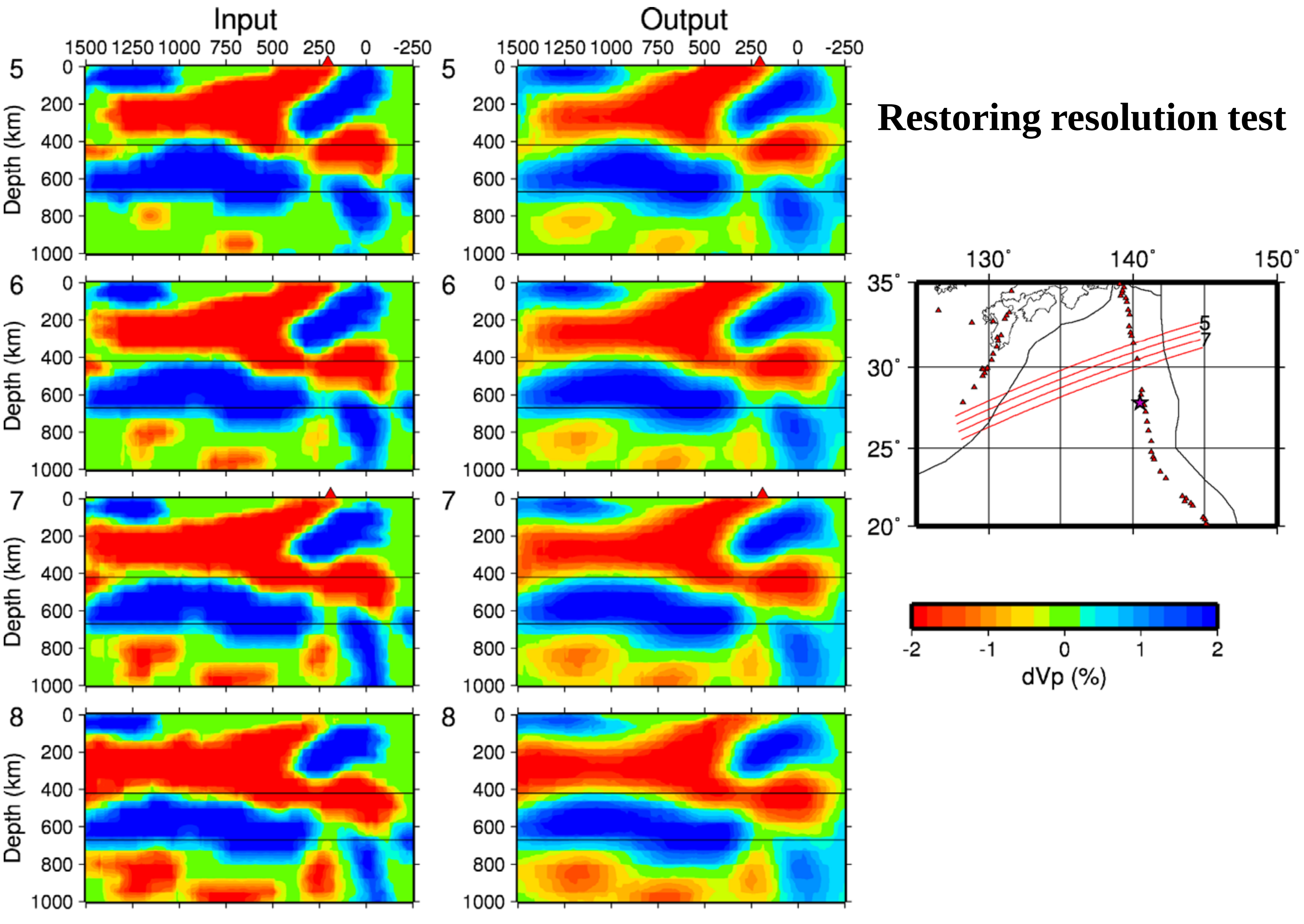

Fig. S10 (continued).

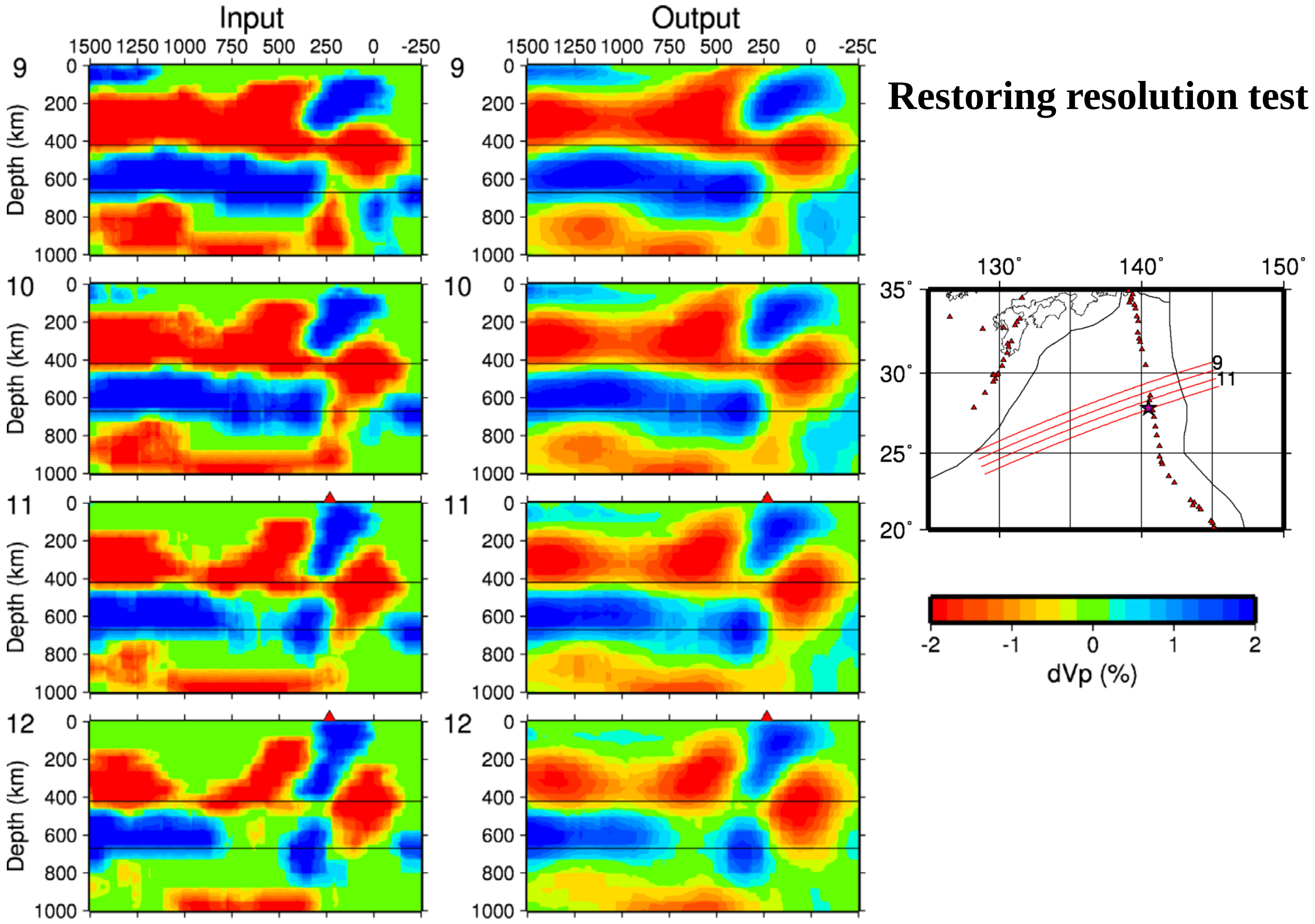

Fig. S10 (continued).

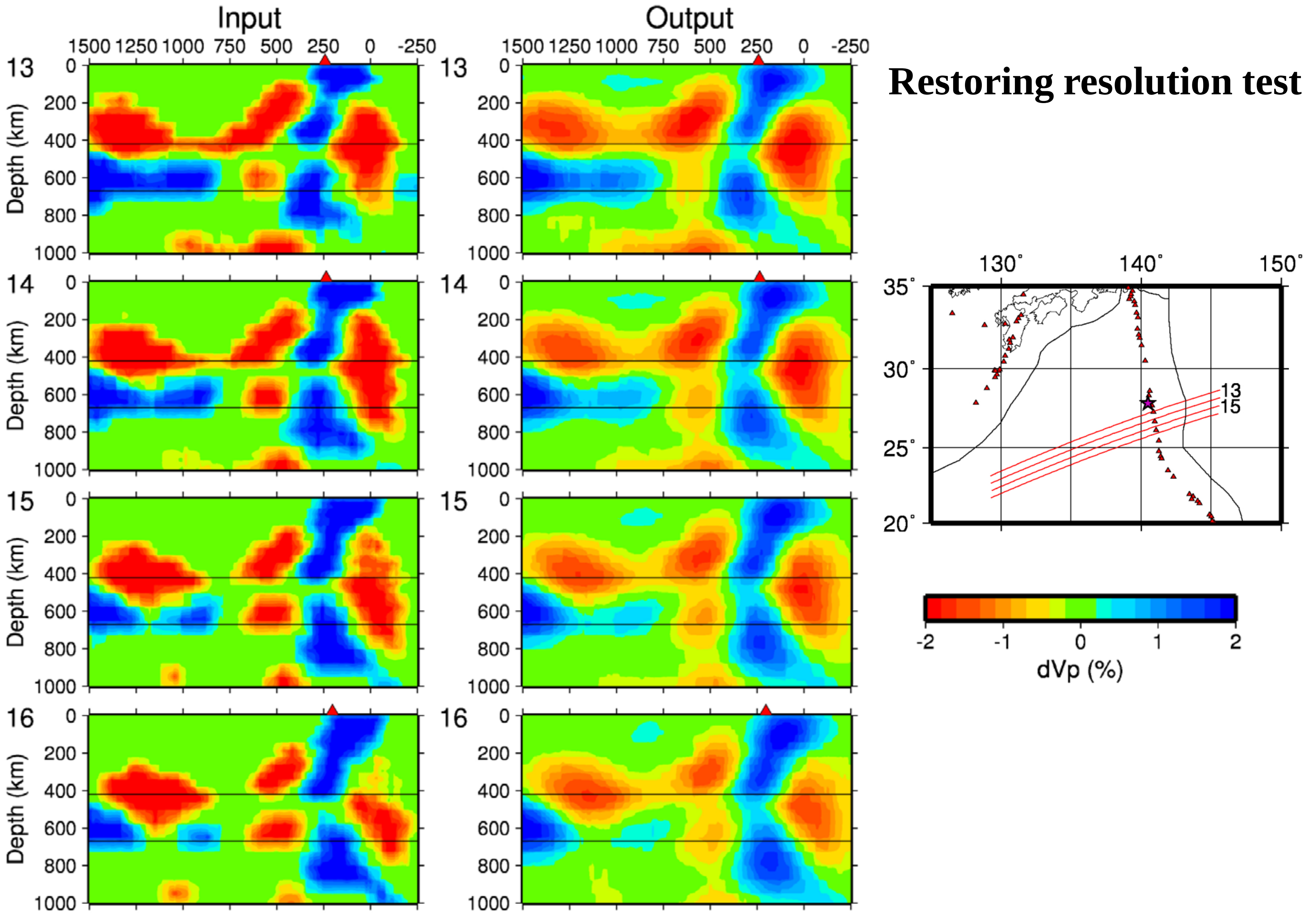

Fig. S10 (continued).

Restoring resolution test

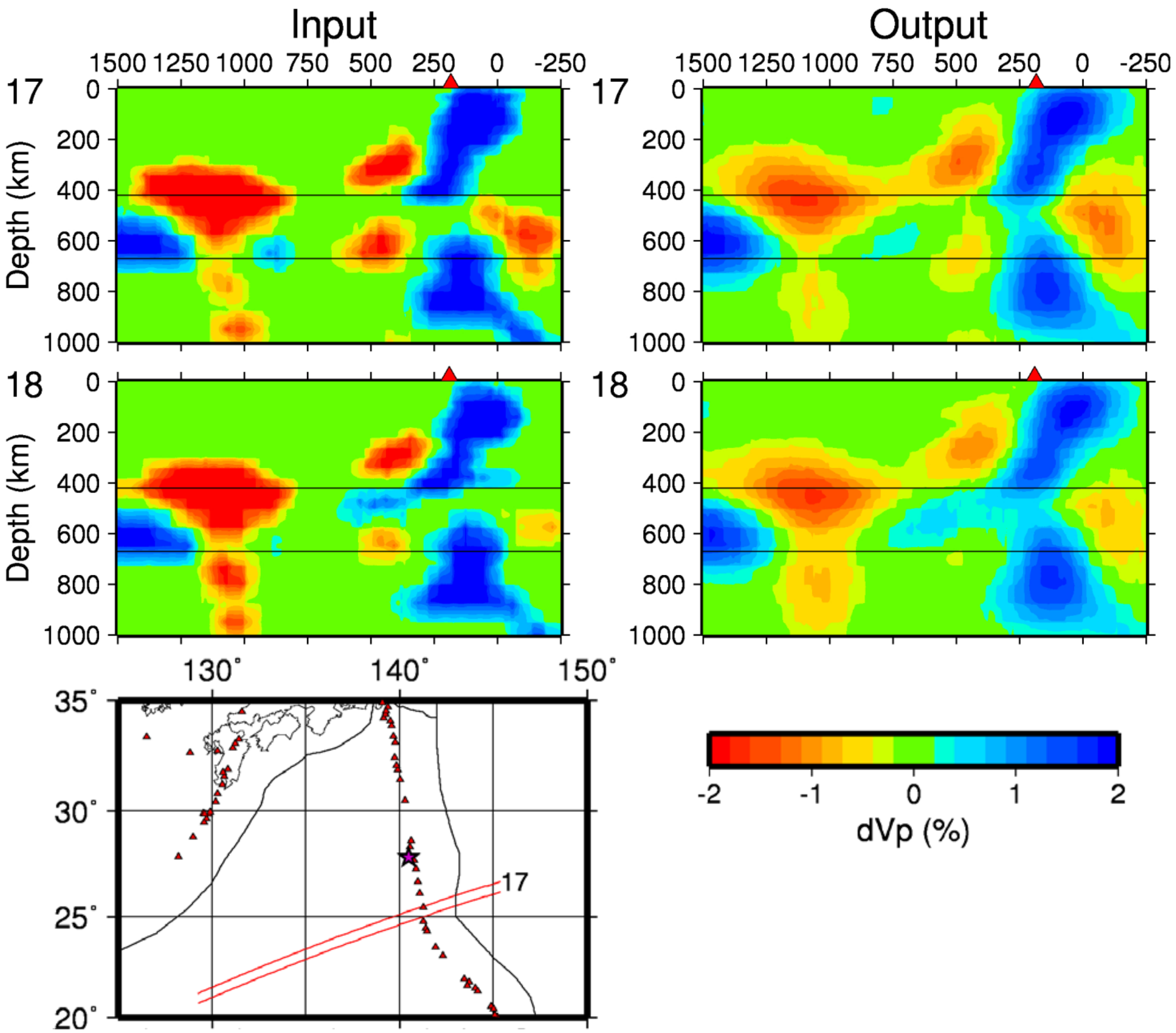

Fig. S10 (continued).

Results of checkerboard resolution tests at 650 km depth

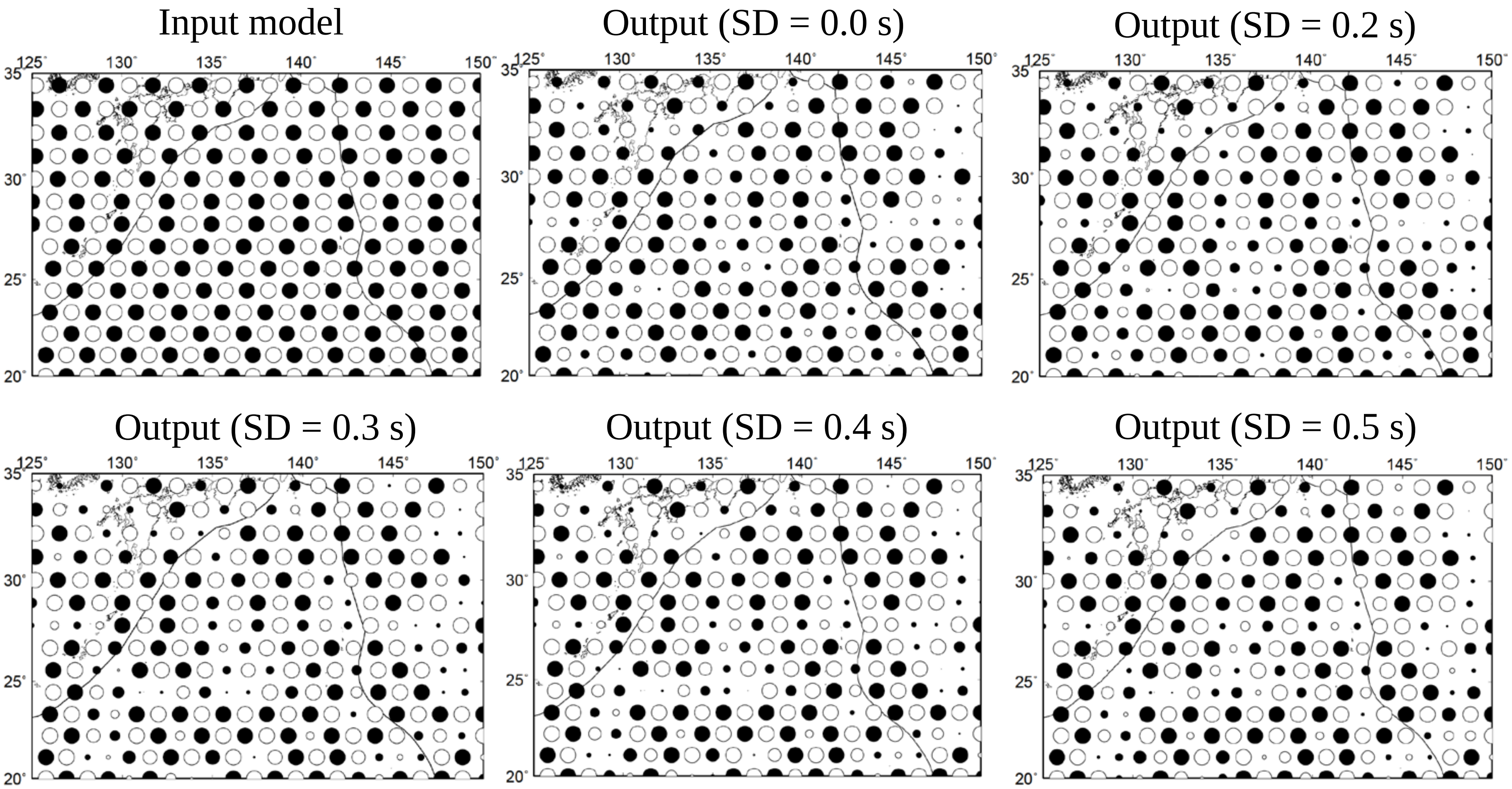

Fig. S11. Results of checkerboard resolution tests at 650 km depth for different levels of random noise. SD means the standard deviation (in second) of random noise added to the synthetic travel-time data before each tomographic inversion. This figure was generated using the Generic Mapping Tools version 4.5.8 (http://gmt.soest.hawaii.edu).
